# Synthesis and Cytotoxic Evaluation of a Series of 2-Amino-Naphthoquinones against Human Cancer Cells

**DOI:** 10.3390/molecules190913188

**Published:** 2014-08-26

**Authors:** Thiago A. P. de Moraes, Maria J. S. Filha, Celso A. Camara, Tania M. S. Silva, Bruno M. Soares, Igor S. Bomfim, Claudia Pessoa, George C. Ximenes, Valdemiro A. Silva Junior

**Affiliations:** 1 Departamento de Morfologia e Fisiologia Animal-UFRPE, Universidade Federal Rural de Pernambuco-UFRPE, Rua Dom Manoel de, s/n, Dois Irmãos, Recife, Pernambuco PE 52171-900, Brazil; E-Mails: thiagomoraes2001@bol.com.br (T.A.P.M.); george.ximenes@terra.com.br (G.C.X.); 2 Departamento de Ciencias Moleculares-UFRPE, Universidade Federal Rural de Pernambuco-UFRPE, Rua Dom Manoel de Medeiros, s/n, Dois Irmãos, Recife, Pernambuco PE 52171-900, Brazil; E-Mails: sarmentomaria@yahoo.com.br (M.J.S.F.); taniasarmento@dcm.ufrpe.br (T.M.S.S.); 3 Departamento de Farmacologia e Fisiologia, Universidade Federal do Ceará-UFC, Rua Coronel Nunes de Melo, 1127, Rodolfo Teófilo, CP 3157, Fortaleza, Ceará CE 60430-270, Brazil; E-Mails: brunomsoares@gmail.com (B.M.S.); igorbiotech@gmail.com (I.S.B.); cpessoa@ufc.br (C.P.)

**Keywords:** naphthoquinones, aminoacid naphthoquinones, cytotoxicity, cancer cells

## Abstract

The cytotoxicity of a series of aminonaphthoquinones resulting from the reaction of suitable aminoacids with 1,4-naphthoquinone was assayed against SF-295 (glioblastoma), MDAMB-435 (breast), HCT-8 (colon), HCT-116 (colon), HL-60 (leukemia), OVCAR-8 (ovarian), NCI-H358M (bronchoalveolar lung carcinoma) and PC3-M (prostate) cancer cells and also against PBMC (peripheral blood mononuclear cells). The results demonstrated that all the synthetic aminonaphthoquinones had relevant cytotoxic activity against all human cancer lines used in this experiment. Five of the compounds showed high cytotoxicity and selectivity against all cancer cell lines tested (IC_50_ = 0.49 to 3.89 µg·mL^−1^). The title compounds were less toxic to PBMC, since IC_50_ was 1.5 to eighteen times higher (IC_50_ = 5.51 to 17.61 µg·mL^−1^) than values shown by tumour cell lines. The mechanism of cell growth inhibition and structure–activity relationships remains as a target for future investigations.

## 1. Introduction

Approximately 518,510 new cases of cancer were estimated for Brazil between 2012 and 2013, including cases of non-melanoma skin cancer, which is the most common type for both men and women (134,000 new cases), followed by prostate (60,000), female breast (53,000), colon and rectum (30,000), lung (27,000), stomach (20,000) and cervix (18,000) [1].

Quinones and their derivatives are substances naturally obtained or synthesized in the laboratory [2] with multiple biological functions in the metabolic cycles of various organisms [3,4]. The major natural sources are higher plants, arthropods, fungi, lichens, bacteria, algae and viruses. They are classified in accordance with the aromatic system present in their structures; naphthoquinones are related to the naphthalenic ring. Several studies have reported the antitumor activity of naphthoquinones due to reduction in the growth of tumor cells lines when combined with other substances [5]. Among the natural naphthoquinones lapachol (2-hydroxy-3-[3-methylbutenyl]-1,4-naphthoquinone) is found in the bark and wood of *Tabebuia* sp and *Tecoma* sp. It has anti-inflammatory, analgesic, antimalarial, antischistosomal, antiviral, antitrypanosomal, leishmanicide, antifungal, anticancer and antiulcerative activities [6,7,8]. β-Lapachone, an *ortho*-naphthoquinone, has been widely studied due to its selective effects on tumor cell lines. Several mechanisms of action related to the naphthoquinone moiety are observed in a dose and time-dependent manner. The cell growth inhibition may occur due to apoptosis, topoisomerase II-α inhibition, oxidative stress and others [5]. The inhibitory action of β-lapachone on α-DNA polymerase provides great potential for chemotherapeutic use against cancer, particularly prostate cancer [3]. Lee and some co-workers [9] demonstrated that β-lapachone, present in *T. avellanedae*, was able to decrease the expression of protein and RNA of COX-2 in a concentration-dependent manner. Furthermore, β-lapachone also inhibited PGE2 production in DU145 (human prostatic carcinoma) cells. The inhibition of the PGE2 and COX-2 expression confirmed that β-lapachone inhibited growth and induces apoptosis in prostatic carcinoma.

The naphthoquinones effectively reduce tumor cell growth in MCF-7 human breast cancer cells when associated with thiosemicarbazone (1,2-naphthoquinone-2-thiosemicarbazone, NTQS) and its metal complexes (Cu^II^, Pd^II^, and Ni^II^), with Ni-NQTS being the most effective among the complexes studied that exerts an antagonizing effect on topoisomerase II activity [10].

Several studies have reported the importance of the antitumor activity of naphthoquinones. β-Lapachone stands out in this field and its biological activity justifies continuing studies with naphthoquinones and their derivatives, such as 2-phenylnaphthoquinone-β-lapachone and esters, activators of oxidative stress agents and topoisomerase II inhibitors, respectively [11,12].

The high incidence of cancer has affected various segments of society; in recent years this fact has been correlated with socio-economic and cultural conditions. Cancer has been the major cause of the high number of deaths as well as mutilations and disabilities affecting the population considerably [1]. Thus, the proposal of this work was to test *in vitro* ten synthetic amino acids linked to the naphthoquinone nucleus in different cancer human cell lines.

## 2. Results and Discussion

**Scheme 1:**
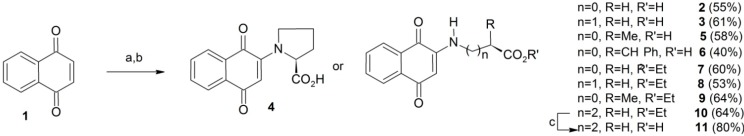
Synthesis of 2-*N*-amino-naphthoquinones **2**–**11**.

The title compounds were obtained in an one-pot Michael nucleophilic 1,4-addition procedure (Scheme 1) from commercially available 1,4-naphthoquinone (**1**) and suitable α- and other amino acids or esters thereof [13,14]. Thus, compounds **2**–**11** were obtained from direct displacement with suitable amino acids or the corresponding ethyl esters in the presence of a base in polar protic or aprotic solvents at room temperature (see Experimental for details). The overall yields of the obtained products ranged from 30% to 64%. All compounds were completely identified using conventional spectroscopic procedures.

*Reagents and Conditions*: (**a**) aminoacid, KOH (aq), NMP, r.t., 1–20h (**2**–**6**); (**b**) amino acid ethyl ester hydrochloride, KOH (aq), MeOH-DMSO, r.t., 10 min (**7**–**10**); (**c**) LiOH, THF (aq), 1 h, r.t. (**11**).

The MTT assay showed the cytotoxic activity of new aminonaphthoquinones (ANPQs) with their respective percentages of inhibition (Table 1). Although the title compounds are not new entities, as far as we know they are being subjected herein to a consistent cytotoxic screening for the first time. Compounds **2** and **6** were previously tested for antimycobacterial activity [15], **7** as a cysteine protease inhibitor [16]. Compound **2** was also tested as a molluscicide [3] and for fungitoxicity [17].

Regarding the human ileocecal adenocarcinoma cell line (HCT-8), the compounds **7**, **6**, **5**, **2** and **9** showed the highest percentage of inhibition of cell growth. Among these samples, **7** had higher mean percentage (99.51%) inhibition against HCT-8, when compared with **6** (86.13%), **5** (84.08%) and **9** (75.36%). Among all tested compounds, **10** had the lowest average percentage of cell growth inhibition, 50% lower than the more efficient compound **7**. The human breast carcinoma cells (MDA-MB-435) showed the highest percentage inhibition of growth when exposed to **2**, **6**, **5**, **9** and **7**. On the other hand, **10** had lower mean percentage of cell growth inhibition (11.13%). The human glioblastoma multiforme cell line (SF-295) showed a percentage inhibition of cell growth of the 99.50% to **7**. When compared with other ANPQs, **7** was indicated to be between 13%–24% more efficient than **6**, **5**, **2** and **9**. In contrast, **8** showed lowest average percentage of cell growth inhibition (32.71%). Table 2 shows cytotoxic activity of the most effective compounds studied herein with their respective half maximal inhibitory concentration (IC_50_) values. It was demonstrated values from 0.49 to 3.11 µg·mL^−1^ showing week selectivity among five tumor cell lines.

**Table 1:** Percentage of inhibition of cell growth (IC%) of the amino-naphthoquinones (ANPQ) at 5 µg/mL in three tumour cell lines from three independent experiments. Values are expressed as mean ± SDM.

	HCT-8	GI%	MDAMB-435	GI%	SF-295	GI%
ANPQ	average	SD	Average	SD	average	SD
2	81.65	3.15	100.00	0.06	81.13	3.26
3	56.04	0.54	26.36	33.11	55.95	0.15
4	59.06	57.01	36.74	70.56	59.33	56.49
5	84.08	1.18	100.00	0.12	83.79	1.12
6	86.13	2.41	100.00	0.09	86.06	2.60
7	99.51	1.28	100.00	0.15	99.50	1.22
8	28.17	17.64	11.67	53.27	32.71	17.12
9	75.36	0.15	100.05	0.22	75.07	0.20
10	49.57	7.03	59.11	12.59	49.21	6.52
11	61.36	8.94	11.13	36.74	61.21	8.61

**Table 2:** Cytotoxicity (IC_50_) of the aminonaphthoquinones (ANPQ) in eight cell lines tumour and normal cell line (PBMC) after 72h exposure, obtained by nonlinear regression for all cell lines from three independent experiments. The values are presented µg·mL^−1^/µmol·L^−1^ and their respective confidence intervals (95% CI).

	HCT-8	MDAMB-435	SF-295	HL-60	HCT-116	OVCAR-8	NCI-H358M	PC3-M	PBMC
2	0.89/3.84	1.21/5.23	0.57/2.46	0.7/3.02	1.31/5.66	3.89/16.78	>5/21.57	>5/21.57	5.51/23.77
	(0.81–0.99)	(0.87–1.69)	(0.41–0.77)	(0.49–1.02)	(1.12–1.54)	(3.52–4.30)			(4.27–7.1)
5	0.83/3.38	0.49/1.99	0.63/2.56	1.05/4.27	1.11/4.52	2.36/9.61	0.90/3.36	1.61/6.55	7.34/29.89
	(0.71–0.96)	(0.42–0.58)	(0.52–0.76)	(0.87–1.26)	(0.94–1.29)	(1.92–2.91)	(0.64–1.29)	(1.46–1.78)	(6.15–8.7)
6	1.59/4.94	1.46/4.53	0.65/2.01	1.31/4.07	2.15/6.67	1.87/5.81	0.82/2.54	1.70/5.28 )	12.18/37.84
	(1.19–2.14)	(1.14–1.88)	(0.52–0.81)	(0.98–1.75)	(1.91–2.42)	(1.62–2.16	(0.62–1.10)	(1.30–2.21	(10.16–14.6)
7	1.84/7.09	1.33/5.12	0.83/3.19	1.24/4.77	2.13/8.20	2.00/7.70	0.64/2.46	1.63/6.28	9.00/34.67
	(1.64–2.06)	(1.05–1.17)	(0.72–0.95)	(1.12–1.37)	(1.76–2.57)	(1.70–2.35)	(0.48–0.85)	(1.37–1.94)	(7.73–10.51)
9	1.33/4.86	1.18/4.31	1.68/6.13	1.74/6.35	3.11/11.36	1.88/6.87	0.98/3.58	2.62/9.57	17.61/64.34
	(0.86–2.07)	(0.88–1.60)	(1.25–2.27)	(1.39–2.17)	(2.61–3.72)	(0.90–3.93)	(0.72–1.33)	(2.22–3.10)	(15.0–20.68)
Dox	0.07/0.13	0.48/0.83	0.23/0.83	0.02/0.03	0.01/0.02	0.26/0.45	0.9/1.65	0.20/0.36	0.23/0.42
	(0.03–0.12)	(0.34–0.66)	(0.19–0.25)	(0.01–0.02)	(0.01–0.02)	(0.17–0.31)	(0.6–1.6)	(0.17–0.23)	(0.10–0.38)

According to the results of Table 2, compound **2** was more efficient against human glioblastoma multiforme cell line (SF-295) and human promyelocytic leukemia cell line (HL-60) with IC_50_ of the 0.57 and 0.7 µg·mL^−1^, respectively. The compound **9** showed 1.18 and 1.33 µg·mL^−1^ cytotoxic activity (IC_50_) to human breast carcinoma cells (MDAMB-435) and ileocecal adenocarcinoma cell line (HCT-8), respectively. The best result observed for **6** and **7** was related to SF-295 with IC_50_ values of 0.65 and 0.83 µg·mL^−1^, respectively. However, another tumor cells lines had an average IC_50_ range from 1.31 to 2.15 µg·mL^−1^. On the other hand, **5** was efficient toward HCT-8, MDAMB-435 and SF-295, with IC_50_s lower than 1.0 µg·mL^−1^. Table 2 also lists values from 0.64 to >5.0 µg·mL^−1^ showing weak selectivity among the OVCAR-8, NCI-H358M and PC3-M cell lines. Nevertheless, human broncho-alveolar lung carcinoma (NCI-H358M) was responsive to all ANPQs with average IC_50_ values ranging from 0.64 and 0.98 µg·mL^−1^, except for **2** that showed an IC_50_ over 5 µg·mL^−1^. The PBMC showed low sensitivity to APQ’s with average IC_50_ values that ranged from 5.51 to 17.61 µg·mL^−1^.

According to results, only **2**, **9**, **6**, **5**, **7** showed cytotoxicity against the cancer cell lines tested. It is noteworthy that ANPQs were less toxic to peripheral blood mononuclear cells, since the IC_50_ was 1.5 to eighteen times higher than the values shown by the cancer cell lines.

Cell culture is an important tool to study the cytotoxicity of compounds with potential therapeutic activity [18]. Several molecules, which have quinone moieties show antiproliferative effects on cancer cell growth. The cytotoxic activity of quinones is related to inhibition of electron transporters, uncoupling of oxidative phosphorylation, ROS generation, protein adduct formation, especially with enzyme SH groups, and DNA damage [19] Silva Jr. *et al.* [20] demonstrated that naphthoquinones and compounds derived from β-lapachone have activity (IC_50_ values below 2 µM) against cancer cell lines. Biflorin, a naphthoquinone isolated from typical *Capraria biflora* L., inhibited the proliferative activity of the five tested cancer cell lines, with better results against intestinal adenocarcinoma HCT-8 [21]. All compounds in this study inhibited the proliferative activity of the adenocarcinoma cell line (HCT-8) with average IC_50_values that ranged from 0.83 to 1.84 µg·mL^−1^. However, it is noteworthy that compound **5** (IC_50_ 0.83 µg·mL^−1^) was as potent as biflorin (IC_50_ 0.88 µg·mL^−1^).

The MDAMB-435 **(**human breast carcinoma) tumor cell line showed great responsiveness when exposed to ANPQs **5** and **9**. Montenegro *et al* [22] evaluated the cytotoxic activity of nine naphthoquinones in five tumor cell lines, obtaining a range from 3.8 to 28.7 µg·mL^−1^ to MDAMB-435 cells. The compounds used in this experiment were more efficient against MDAMB-435 cells since **5** and **9** showed an average range from 0.49 and 1.18 µg·mL^−1^, respectively.

The human glioblastoma multiforme cell line (SF-295) was very sensitive to all ANPQs with average IC_50_ values ranging from 0.57 to 1.68 µg·mL^−1^ (2.46–6.13 µmol·L^−1^). Cardioquinones isolated from the roots of *Cordia polycephala* were evaluated against four human cancer cell lines: HCT-8 (colon), HL-60 (leukemia), MDAMB-435 (melanoma) and SF295 (glioblastoma), showing IC_50_ values in the range of 1.2 to 11.1 µmol·L^−1^ [19]. However, the glioblastoma multiforme cell line (SF-295) showed an IC_50_ of the 1.8 µmol·L^−1^ related to cardioquinones whereas the ANPQs showed an IC_50_ 68% lower than the better cardioquinone used against SF-295 cell line. Naphthoquinones derived from cardioquinones [19] and biflorin [21] had cytotoxic activity to HL-60 tumor cell line with the better IC_50_ values been 1.2 and 1.95 µmol·L^−1^. In this experiment, all compounds were more cytotoxic than the previously cited naphthoquinones; however, it is noteworthy that compound **2**, with an IC_50_ of 3.02 µmol·L^−1^, is the best of all.

Naphthoquinones from *Alkanna tinctoria* (L.) Tausch showed antiproliferative effects on human colorectal cancer cells with minimum inhibitory concentration (IC_50_) values ranging from 2.38 to 4.76 µmol·L^−1^ for HCT-116 cells [23]. The aminonaphthoquinones showed better antiproliferative activity to human colorectal cancer cells since their IC_50_ values were very close to those previously cited (1.11 to 3.11 µmol·L^−1^). The platinum (II) complexes of 3-(aminomethyl)naphthoquinone Mannich bases [24] exhibited significant cytotoxic activity against the same cell lines tested in this experiment, although they were only moderately active against the PC-3M cell line. On the other hand, the ANPQs showed a high activity (IC_50_ values 5.28 to 9.57 µmol·L^−1^) against PC-3M, except for **5** (IC_50_ values > 21.57 µmol·L^−1^). 

The low toxicity of ANPQs (IC_50_ 23.77–64.34 µmol·L^−1^) tested against PBMC shows that these compounds are selective for the studied cancer cells. The cytotoxic activity of juglone and its 5-*O*-methyl derivative showed an IC_50_ of 0.69 µg·mL^−1^ (3.7 µmol·L^−1^) against PBMC (Montenegro *et al.* [22]). A series of 2,3-diyne-1,4-naphthoquinone derivatives synthesized from 2,3-dibromo-1,4-naphthoquinone and various functionalized terminal alkynes using Pd-catalyzed Sonogashira cross-coupling reactions showed considerably high toxicity against PBMC (IC_50_ 0.045–0.970 µmol·L^−1^) [25].

## 3. Experimental Section

### 3.1. General Information

The ^1^H- and ^13^C-NMR spectra were recorded on a Varian Mercury-200, 300 or 400 MHz apparatus with commercial available deuterated solvents (Aldrich, St. Louis, MI, USA and Cambridge Isotopes Laboratories, Tewksbury, MA, USA). The chemical shift values (δ) were expressed in parts per million (ppm) and the coupling constants (*J*) in Hertz (Hz). The infrared spectra (FT-IR) were obtained in Bomem Series MB-100 and Varian 640-IR spectrophotometer with a Germanium Golden Gate ATR accessory. The values for the absorptions are reported in wave numbers, using as unit the reciprocal centimeters (cm^−1^). The melting point was carried out at a Bio San PFM apparatus and not corrected. The products were purified by adsorption column chromatography, using silica gel 60 (230–400 mesh ASTM, Merck, Whitehouse Station, NJ, USA) as stationary phase and commercial solvents (Dinamica-Jandira, São Paulo, Brazil,Cinética,-Diadema, São Paulo, Brazil, Merck, Tedia, Fairfield, OH, USA, among others) as mobile phase. Analytical thin layer chromatography (TLC) were performed using silica gel 60 (F_254_-Merck) plates. The visualization of the spots was made in TLC with the aid of UV lamp (254 and 365 nm). Elemental analyses were carried out in a CA EA1110 CHNS-O analyzer.

### 3.2. Synthesis

#### 3.2.1. General Procedure for the Preparation of Compounds **2**–**6**

1,4-Naphthoquinone (**1**, 14 mmol) dissolved in NMP (20 mL) was placed in an Erlenmeyer flask and a suitable natural L-aminoacid or equivalent (15 mmol) dissolved in water (10 mL) was added at room temperature. The resulting mixture was immediately added to a stirred solution of 3 N KOH (5 mL). This mixture was left under stirring and the reaction completion was monitored by TLC. After filtering under vacuum the mixture was acidified with HCl (10%), and the precipitate formed was filtered under vacuum and washed with cooled dichloromethane. The residue was chromatographed on a silica gel column eluted with a CH_2_Cl_2_–MeOH gradient (100:0 to 90:10 v/v) to afford the target compounds.

*2N-(1,4-Dioxo-1,4-dihydronaphthalen-2-yl)acetic acid* (**2**). Obtained as a red solid in 55% yield from glycine after 2 h reaction, mp 168–170 °C (lit.: decomposition at 197–199 °C [26]); IR (ATR) ν_max_/cm^−1^ 3341, 1729, 1683, 1607, 1558, 1505, 1301; ^1^H-NMR (DMSO-*d*_6_, 300 MHz): 12.43 (sl, 1H), 7.97 (dd, 1H, *J =* 7.5/1.6 Hz), 7.90 (dd, 1H, *J =* 7.5/1.6 Hz), 7.82 (td, 1H, *J =* 7.5/1.8 Hz), 7.74 (td, 1H, *J =* 7.5/1.5 Hz), 7.45 (t, 1H, N-H, *J =* 6.0 Hz), 5.62 (s, 1H), 3.98 (d, 2H, *J =* 6.0 Hz), ^13^C-NMR (DMSO-*d*_6_, 75 MHz): 181.7, 181.3, 170.2, 148.2, 134.9, 132.9, 132.4, 130.2, 125.9, 125.4, 100.7, 43.5. Anal. Calcd. for C_12_H_9_NO_4_: C, 62.34; H, 3.92; N, 6.02. Found: C, 63.82; H, 5.44; N, 5.38.

*3N-(1,4-Dioxo-1,4-dihydronaphthalen-2-yl)propanoic acid* (**3**). Obtained as an orange solid in 61% yield from β-alanine after 2 h reaction, mp 198–200 °C (lit.: 206–207 °C [27]); IR (ATR) ν_max_/cm^−1^ 3446, 3390, 1718, 1670, 1592, 1560, 1512, 1251; ^1^H-NMR (DMSO-*d*_6_, 300 MHz) 12.43 (sl, 1H), 7.92 (d, 1H *J* = 7.5 Hz), 7.90 (d, 1H, *J* = 7.5 Hz), 7.77 (dd, 1H *J* = 7.5/1.0 Hz), 7.67 (dd, 1H *J* = 7.5/1.0 Hz), 7.43 (t, 1H, N-H, *J* = 6.0 Hz), 5.67 (s, 1H), 3.37 (dd, 2H, *J* = 6.0/6.9 Hz), 2.59 (t, 2H, *J* = 6.9 Hz); ^13^C-NMR (DMSO-*d*_6_, 75 MHz): 181.4, 181.4, 172.8, 148.3, 134.8, 133.1, 132.2, 130.3, 125.9, 125.4, 99.7, 37.9, 32.2. Anal. Calcd. for C_13_H_11_NO_4 × _H_2_O: C, 59.31; H, 4.98; N, 5.32. Found: C, 58.89; H, 5.37; N, 5.28.

*N-(1,4-Dioxo-1,4-dihydronaphthalen-2-yl)-L-proline* (**4**). Obtained as an orange solid in 58% yield from L-proline after 1 h reaction, mp 164–167 °C (lit.: decomposition at 165–168 °C [14]); IR (ATR) ν_max_/cm^−1^ 1724, 1678, 1590, 1541, 1475. 1296; ^1^H-NMR (DMSO-*d*_6_, 400 MHz): 12.43 (sl, 1H), 7.90 (d, 2H, *J* = 7.6 Hz), 7.80 (t, 1H, *J* = 7.6 Hz), 5.74 (s, 1H), 7.71 (t, 1H, *J* = 7.6/7.6 Hz) , 4.98 (m, 1H), 3.34 (m, 2H), 2.27 (m, 1H), 2.06 (m, 1H), 1.95 (m, 1H), 1.88 (m, 1H); ^13^C-NMR (DMSO-*d*_6_, 100 MHz): 182.6, 181,1, 173.3, 148.7, 134.3, 132.3, 132.2, 131.2, 126.1, 124.7, 104.8, 62.4, 50.9, 30.9, 21.8. 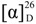 = +84.7 (C = 0.01, DMSO). Anal. Calcd. for C_15_H_13_NO_4_: C, 66.41; H, 4.83; N, 5.16. Found: C, 66.14; H, 4.85; N, 5.35.

*N-(1,4-Dioxo-1,4-dihydronaphthalen-2-yl)-L-alanine* (**5**). Obtained as an orange solid in 58% yield from L-alanine after 3 h reaction, mp 148–150°C (lit.: 139–142 °C [13]); IR (ATR) ν_max_/cm^−1^ 3356, 1722, 1681, 1604, 1556, 1450, 1302; ^1^H-NMR (DMSO-*d*_6_, 300 MHz) 13.14 (s, 1H), 7.98 (d, 1H *J* = 7.5 Hz), 7.91 (d, 1H *J* = 7.5 Hz), 7.81 (t, 1H *J* = 7.5 Hz), 7.72 (t, 1H *J* = 7.5 Hz), 7.27 (d, 1H, N-H, *J* = 7.5 Hz), 5.65 (s, 1H), 4.21 (quint, 1H *J* = 7.2 Hz), 1.45 (d, 3H *J* = 7.2 Hz); ^13^C-NMR (DMSO-*d*_6_, 75 MHz) 181.7, 181.3, 173.1, 147.4, 134.9, 132.7, 132.4, 130.2, 126.0, 125.4, 100.9, 50.1, 16.7. 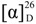 = +14.4 (C = 0.01, DMSO). Anal. Calcd. for C_13_H_11_NO_4_: C, 63.67; H, 4.52; N, 5.71. Found: C, 61.75; H, 4.70; N, 5.70.

*N-(1,4-Dioxo-1,4-dihydronaphthalen-2-yl)-L-phenylalanine* (**6**). Obtained as an orange solid in 60% yield from L-phenylalanine after 24 h reaction, mp 128–130 °C (lit.: dark oil [15]); IR (ATR) ν_max_/cm^−1^ 3338, 3066, 3032, 1722, 1681, 1598, 1556, 1503, 1305; ^1^H-NMR (DMSO-*d*_6_, 400 MHz) 7.93 (d, 1H, *J* = 7.5 Hz), 7.89 (d, 1H, *J* = 7.5 Hz), 7.79 (t, 1H, *J* = 7.5 Hz), 7.69 (t, 1H, *J* = 7.5 Hz), 7.23 (m, 4H), 7.16 (m, 1H), 7.06 (d, 1H, NH, *J* = 8 Hz), 5.72 (s, 1H), 4.48 (q, 1H, *J* = 7.2 Hz), 3.21 (2H, m); ^13^C-NMR (DMSO-*d*_6_, 100 MHz) 181.7, 181.1, 171.7, 147.3, 136.8, 134.9, 132.6, 132.4, 130.0, 129.2, 128.2, 126.6, 125.9, 125.3, 101.1, 55.5, 35.7. Anal. Calcd. for C_19_H_15_NO_4 × _H_2_O: C, 67.25; H, 5.05; N, 4.13. Found: C, 67.87; H, 5.49; N, 3.98. 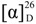 = +85.86 (C = 0.01, DMSO).

#### 3.2.2. General Procedure for the Preparation of Compounds **7**–**10**

1,4-Naphthoquinone (**1**, 14 mmol) dissolved in DMSO (20 mL) and the appropriate ethyl ester amino acid hydrochloride (15 mmol) dissolved in water (10 mL) were mixed in an Erlenmeyer flask at room temperature. The resulting mixture was immediately added to a stirred 3N solution of KOH (5 mL) in MeOH. This mixture was left under stirring and the reaction completion was monitored by TLC. After vacuum filtration the mixture was acidified by means of HCl (10%), and the precipitate formed was vacuum filtered and washed with cooled water. The residue was chromatographed on silica gel column with *n*-hexane-CH_2_Cl_2_ (100:0 to 50:50 v/v) as eluent to afford the target compounds.

*Ethyl 2N-(1,4-dioxo-1,4-dihydronaphthalen-2-yl)acetate* (**7**). Obtained as an yellow solid in 30% yield from ethyl glycinate after 2 h reaction, mp 93–95 °C (lit.: not found [16]); IR (ATR) ν_max_/cm^−1^ 3391, 3050, 2924, 1732, 1673, 1643, 1611, 1222; ^1^H-NMR (CDCl_3_, 200 MHz): 8.05 (dd, 1H, *J* = 7.5/1.4 Hz), 8.02 (dd, 1H, *J* = 7.5/1.4 Hz), 7.69 (td, 1H *J* = 7.5/1.4 Hz), 7.59 (td, 1H *J* = 7.5/1.2 Hz), 6.37 (sl, 1H N-H), 5.62 (s, 1H), 4.25 (q, 2H, *J* = 7.0 Hz), 3.90 (d, 2H, *J* = 5.0 Hz), 1.30 (t, 3H, *J* = 7.0 Hz); ^13^C-NMR (CDCl_3_, 50 MHz) 183.1, 181.1, 168.2, 147.1, 134.7, 133.2, 132.2, 130.4, 126.3, 126.1, 102.0, 62.0, 43.8, 14.1. Anal. Calcd. for C_14_H_13_NO_4_: C, 64.86; H, 5.05; N, 5.40. Found: C, 63.77; H, 5.21; N, 4.15.

*Ethyl 3N-(1,4-dioxo-1,4-dihydronaphthalen-2-yl)propanoate* (**8**). Obtained as an orange solid in 53% yield from ethyl β-aminopropionate after 1 h, mp 120–122 °C (lit.: 123–125 °C [28]); IR (ATR) ν_max_/cm^−1^ 3374, 3355, 3062, 2999, 1730, 1680, 1629, 1607, 1221; ^1^H-NMR (CDCl_3_, 200 MHz) 8.05 (dd, 1H, *J* = 7.5/1.0 Hz), 8.00 (dd, 1H, *J* = 7.5/1.2 Hz), 7.69 (td, 1H, *J* = 7.4/1.6 Hz), 7.57 (td, 1H, *J* = 7.6/1.0 Hz), 6.22 (sbr, 1H, N-H), 5.73 (s, 1H), 4.15 (q, 2H, *J* = 7.0 Hz), 3.50 (q, 2H, *J* = *6*.0 Hz), 2.64 (dd, 2H, *J* = 6.4/8.0 Hz), 1.24 (t, 3H *J* = 7.0 Hz); ^13^C-NMR (CDCl_3_, 50 MHz): 182.9, 181.4, 171.1, 147.6, 134.7, 133.3, 132.0, 130.4, 126.3 , 126.1, 100.7, 61.0, 37.9, 32.6, 14.1. Anal. Calcd. for C_15_H_15_NO_4_: C, 65.92; H, 5.53; N, 5.13. Found: C, 65.78; H, 5.76; N, 4.89.

*Ethyl [(S) 2-(1,4-dioxo-1,4-dihydronaphthalen-2-ylamino)] propanoate* (**9**). Obtained as an orange solid in 64% yield from ethyl L-alanine ester after 2 h reaction, mp 65–68 °C (lit.: not found [26]); IR (ATR) ν_max_/cm^−1^ 3374, 3355, 3062, 2999, 1730, 1680, 1629, 1607, 1221; ^1^H-NMR (CDCl_3_, 200 MHz) 8.01 (dd, 1H, *J* = 7.5/1.2 Hz), 7.99 (dd, 1H, *J* = 7.5/1.2 Hz), 7.69 (td, 1H, *J* = 7.5/1.2 Hz), 7.59 (td, 1H *J* = 7.5/1.2 Hz), 6.30 (sbr, 1H, N-H), 5.62 (s, 1H), 4.18 (q, 2H, *J* = 7.0 Hz), 4.04 (m, 1H), 1.50 (d, 3H, *J* = 7.0 Hz), 1.22 (t, 3H, *J* = 7.0 Hz); ^13^C-NMR (CDCl_3_, 50 MHz) 183.1, 181.3, 171.5, 146.5, 134.6, 133.1, 132.1, 130.3, 126.3, 126.0, 101.8, 61.9, 50.4, 17.4, 14.0. Anal. Calcd. for C_15_H_15_NO_4_: C, 65.92; H, 5.53; N, 5.13. Found: C, 65.32; H, 5.80; N, 4.89.

*Ethyl 4-(1,4-dioxo-1,4-dihydronaphthalen-2-ylamino)butanoate* (**10**). Obtained as an orange solid in 64% yield from ethyl 4-aminobutanoate ester after 2 h reaction, mp 120–122 °C (lit.: not found [29]); IR (ATR) ν_max_/cm^−1^ 3335, 2717, 2637, 1694, 1671, 1618, 1595, 1465; ^1^H-NMR (CDCl_3_, 200 MHz) 8.05 (dd, 1H, *J* = 7.5/1.2 Hz), 8.01 (dd, 1H, *J* = 7.5/1.2 Hz), 7.69 (td, 1H, *J* = 7.5/1.2 Hz), 7.59 (td, 1H *J* = 7.5/1.2 Hz), 6.08 (sbr, 1H, N-H), 5.73 (s, 1H), 4.12 (q, 2H, *J* = 7.0 Hz), 3.24 (q, 1H, *J* = 7.2 Hz), 2.41 (t, 2H, *J* = 7.0 Hz), 1.99 (qui, 2H, *J* = 7.1 Hz), 1.23 (t, 3H, *J* = 7.0 Hz); ^13^C-NMR (CDCl_3_, 50 MHz) 182.8, 181.6, 172.8, 148.0, 134.7, 133.4, 132.0, 130.3, 126.3, 126.1, 100.7, 60.7, 42.0, 31.6, 17.4, 23.1, 14.1. Anal. Calcd. for C_15_H_15_NO_4_: C, 65.92; H, 5.53; N, 5.13. Found: C, 65.71; H, 6.24; N, 4.77.

*4-(1,4-Dioxo-1,4-dihydronaphthalen-2-ylamino)butanoic acid* (**11**). To a mixture of LiOH (30 mg), THF (2 mL) and water (0.5 mL) was added the ester **10a** (100 mg). The mixture was stirred at room temperature and inspected by TLC until complete disappearance of the ester **10** (after 2 h reaction). The mixture was filtered under vacuum, acidified with 10% HCl and an amorphous orange solid filtered off in 80% yield, mp 210–212 ° C (lit.: 210–211 °C [13,26]); IR (ATR) ν_max_/cm^−1^ 3335. 2717, 2637, 1694, 1671, 1618, 1595, 1296; ^1^H-NMR (CDCl_3_, 300 MHz) 12.17 (sbr, 1H), 7.95 (dd, 1H *J* = 7.8/0.9 Hz), 7.93 (dd, 1H *J* = 7.9/0.9 Hz), 7.80 (m, 1H), 7.67 (m, 1H), 5.7 (s, 1H), 3.20 (q, 2H, *J* = 6.6 Hz), 2.30 (t, 2H, *J* = 7.2 Hz), 1.78 (quint, 2H, *J* = 7.2 Hz); ^13^C-NMR (CDCl_3_, 75 MHz): 181.5, 181.3, 174.3, 148.6, 134.8, 133.2, 132.1, 130.4, 125.8, 125.3, 99.3, 41.2, 30.6, 22.6. Anal. Calcd. for C_16_H_17_NO_4 × _H_2_O: C, 62.94; H, 6.27; N, 4.59. Found: C, 61.14; H, 5.63; N, 4.73.

### 3.3. Cytotoxicity against Cancer Cell Lines

This experiment evaluated the cytotoxicity of aminonaphthoquinones (ANPQs) against human cancer cells, *in vitro*. This analysis is part of an initial screening for determination of the potential antitumor of these samples. The human cancer cell strains used were SF-295 (glioblastoma-human), MDAMB-435 (melanoma), HCT-8 (colon), HCT-116 (colon), HL-60 (leukemia), OVCAR-8 (ovarian cancer), NCI-H358M (human bronchoalveolar lung carcinoma) and PC3-M (highly metastatic prostate cancer cell line). All cancer cells were provided by the National Cancer Institute (Rockville, MD, USA) and were cultured in RPMI 1640 supplemented with 10% fetal bovine serum and 1% antibiotics, maintained at 37 °C and atmosphere containing 5% CO_2_. The peripheral blood mononuclear cells (PBMC) were isolated by gradient of density (Ficoll-Hypaque) and cultivated in RPMI 1640 medium, supplemented with bovine foetal serum (20%), antibiotic (1%) and phytohemagglutinin (4%). The cells were maintained at 37 °C and atmosphere containing 5% CO_2_.

The different samples of ANPQs were diluted in pure sterile DMSO. The substances were tested at a concentration of 5 µg·mL^−1^. In order to determine the IC_50_ the substances were tested at increasing concentrations in serial dilutions (5–0.010 µg·mL^−1^). Doxorubicin (5–0.0010 µg·mL^−1^) was used as positive control only for the determination of IC_50_.

Analysis of cytotoxicity by the MTT method has been used in the screening program at the National Cancer Institute of the United States (NCI), which tests over 10,000 samples per year [30]. It is a rapid, sensitive and inexpensive test. It was first described by Mosman [31] as having the ability to analyze the viability and the metabolic state of cells. It is a colorimetric analysis based on conversion of the salt 3-(4,5-dimethyl-2-thiazole)-2,5-diphenyl-2H-tetrazolium bromide (MTT) to formazan-blue by the mitochondrial enzymes found only in metabolically active cells. The study of the cytotoxicity by MTT method allows easily defining the cytotoxicity but not the mechanism of action [32].

The cells were plated at a concentration of 0.1 × 10^6^ cell mL^−1^ to OVCAR-8, NCI-H358M, PC3-M, SF-295 and MDA-MB435 strains, 0.7 × 10^5^ cell mL^−1^ to HCT-8 and HCT-116 strains, 0.3 × 10^6^ to HL-60 strain, 1.0 × 10^6^ cell mL^−1^ to PBMC. The plates were incubated for 72 h at 5% CO_2_ at 37 °C. After, they were centrifuged and the supernatant removed. Then the solution of MTT (tetrazolium salt, 150 µL) was added, and the plates were incubated for 3 h. The absorbance was read after dissolving the precipitate with 150 µL of pure DMSO in a spectrophotometer plate at 595 nm.

### 3.4. Statistical Analysis

The experiments were analyzed by mean ± standard deviation of the mean (SDM) of the percentage of cell growth inhibition using GraphPad Prism. 

## 4. Conclusions

Our findings have demonstrated the cytotoxic potential against human cancer lines of a series of synthetic aminoacids conjugated with a naphthoquinone nucleus. As shown by various tumor cell lines, only five samples (**2**, **9**, **6**, **5** and **7**) showed high cytotoxic activity and selectivity against the eight human cancer cell lines tested. Furthermore, the aminonaphthoquinones in this study were less toxic to peripheral blood mononuclear cells, since the IC_50_s were 1.5 to eighteen times higher than values shown by tumor cell lines. The mechanism of cell growth inhibition and structure–activity relationship of the compounds remains the target of future investigations.

## Supplementary Materials

Supplementary materials can be accessed at: http://www.mdpi.com/1420-3049/19/9/13188/s1.

## Supplementary Files

Supplementary File 1
