# Supplementary material for: Synthesis and Cytotoxic Evaluation of a Series of 2-Amino-Naphthoquinones against Human Cancer Cells
Source: Molecules. 2014 Aug 26;19(9):13188–99. doi: 10.3390/molecules190913188 (PMC6271085; doi:10.3390/molecules190913188)

Suplementary Materials

Figure S1. IR (ATR, cm^{-1}) of compound **2**.

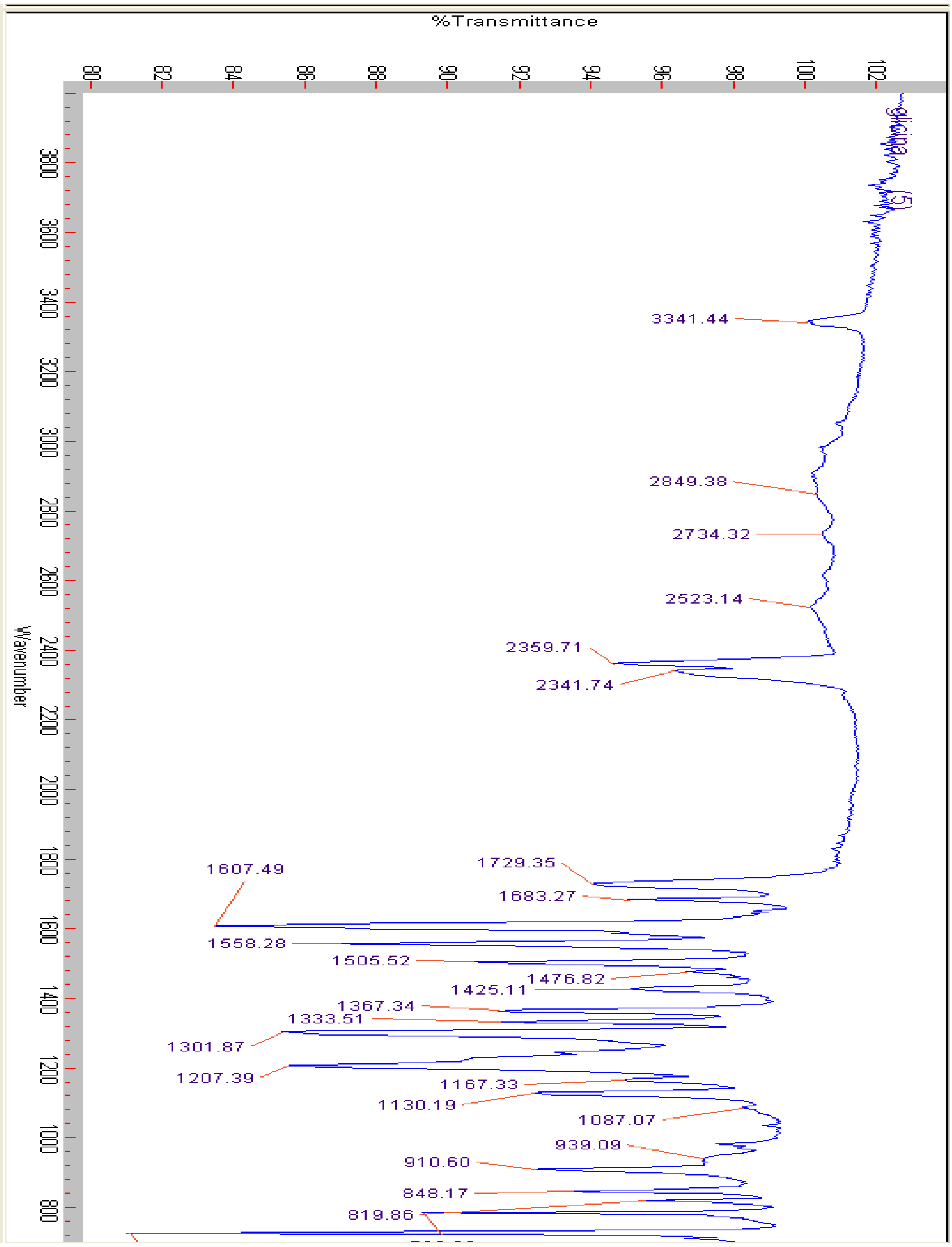

Figure S2. ^{1}H-NMR (δ, DMSO-d_6, 300 MHz) and expansion of **2**.

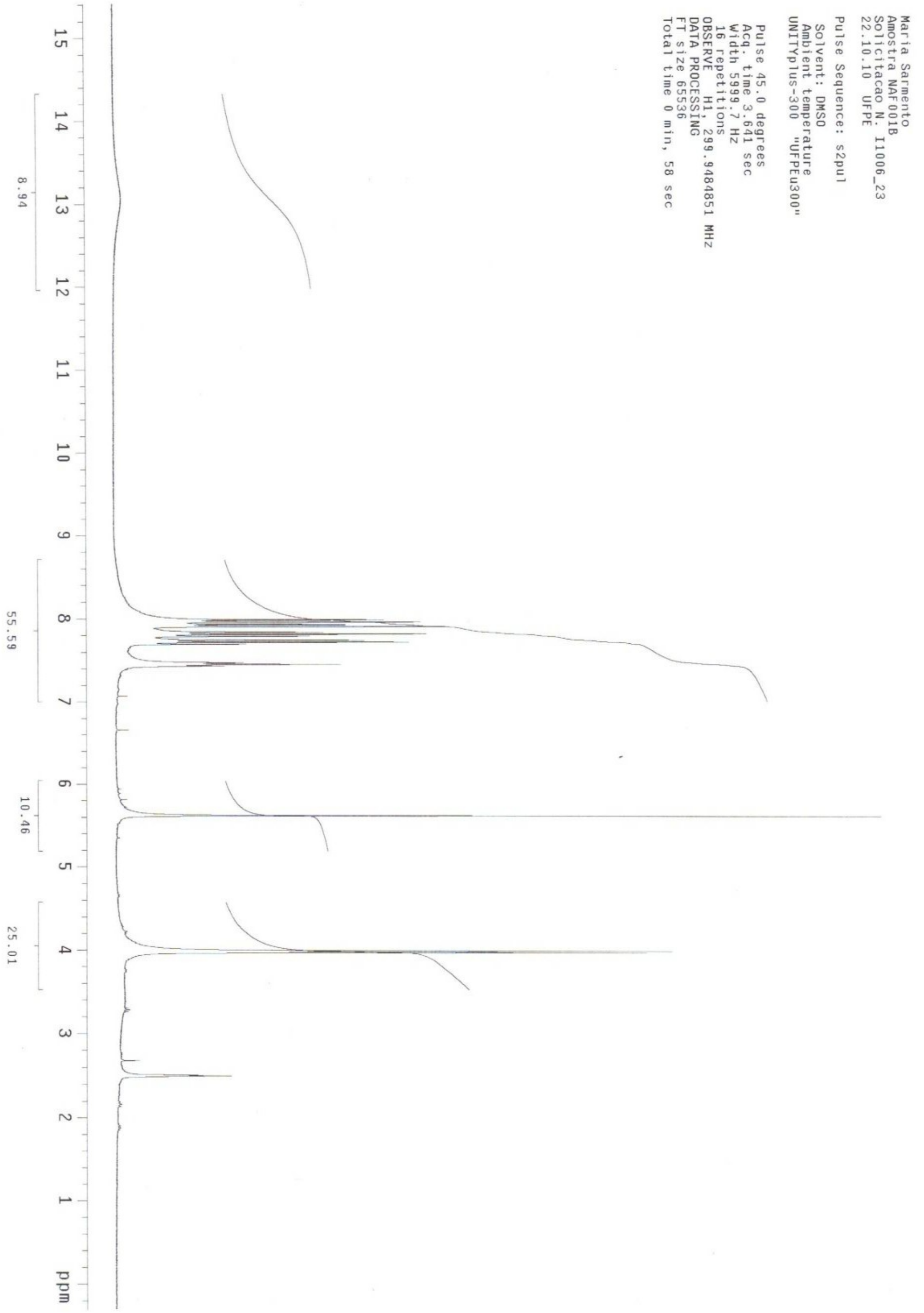

Figure S2. *Cont.*

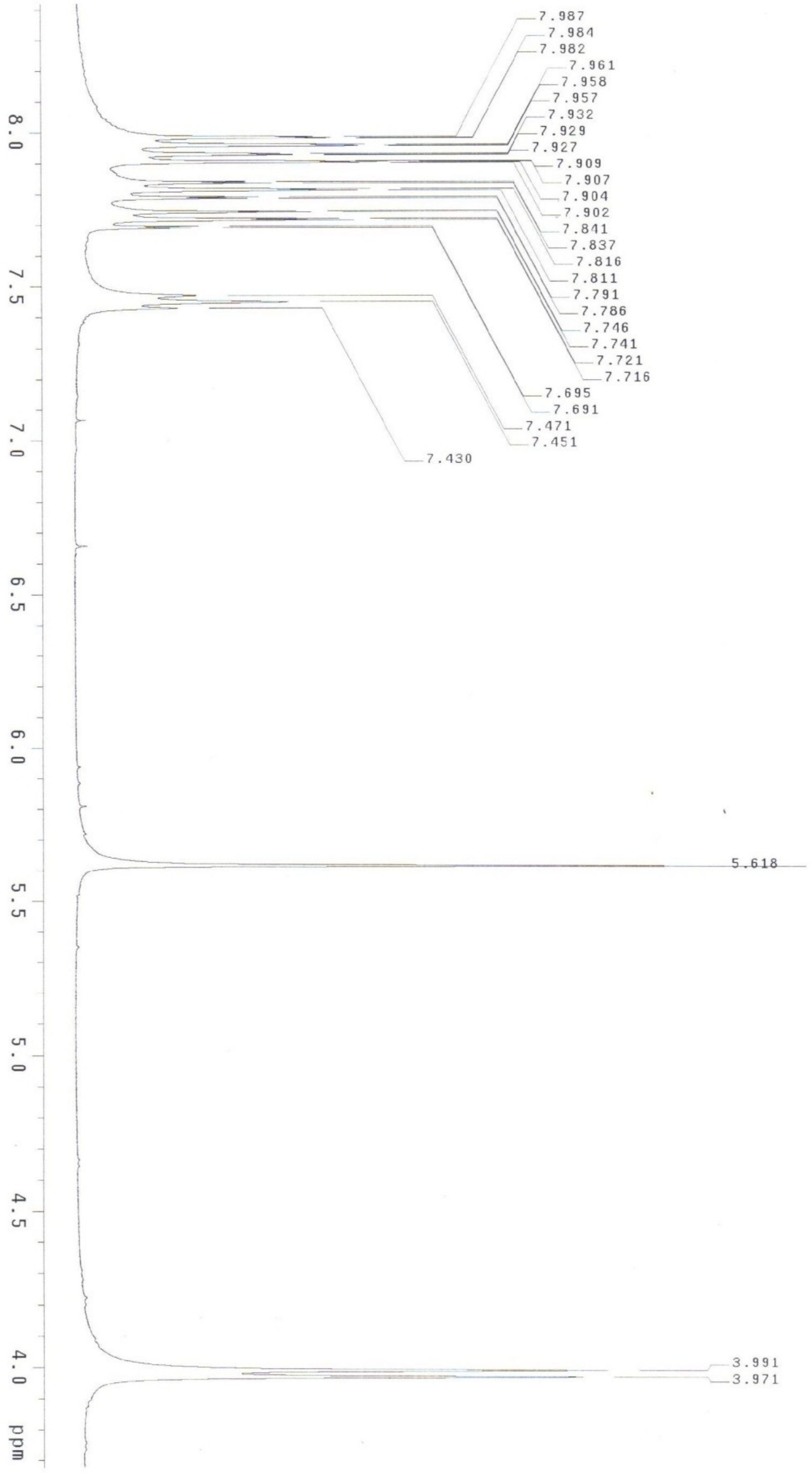

Figure S3. ^{13}C NMR of (DMSO- $_{d6}$, 75 MHz) of **2**.

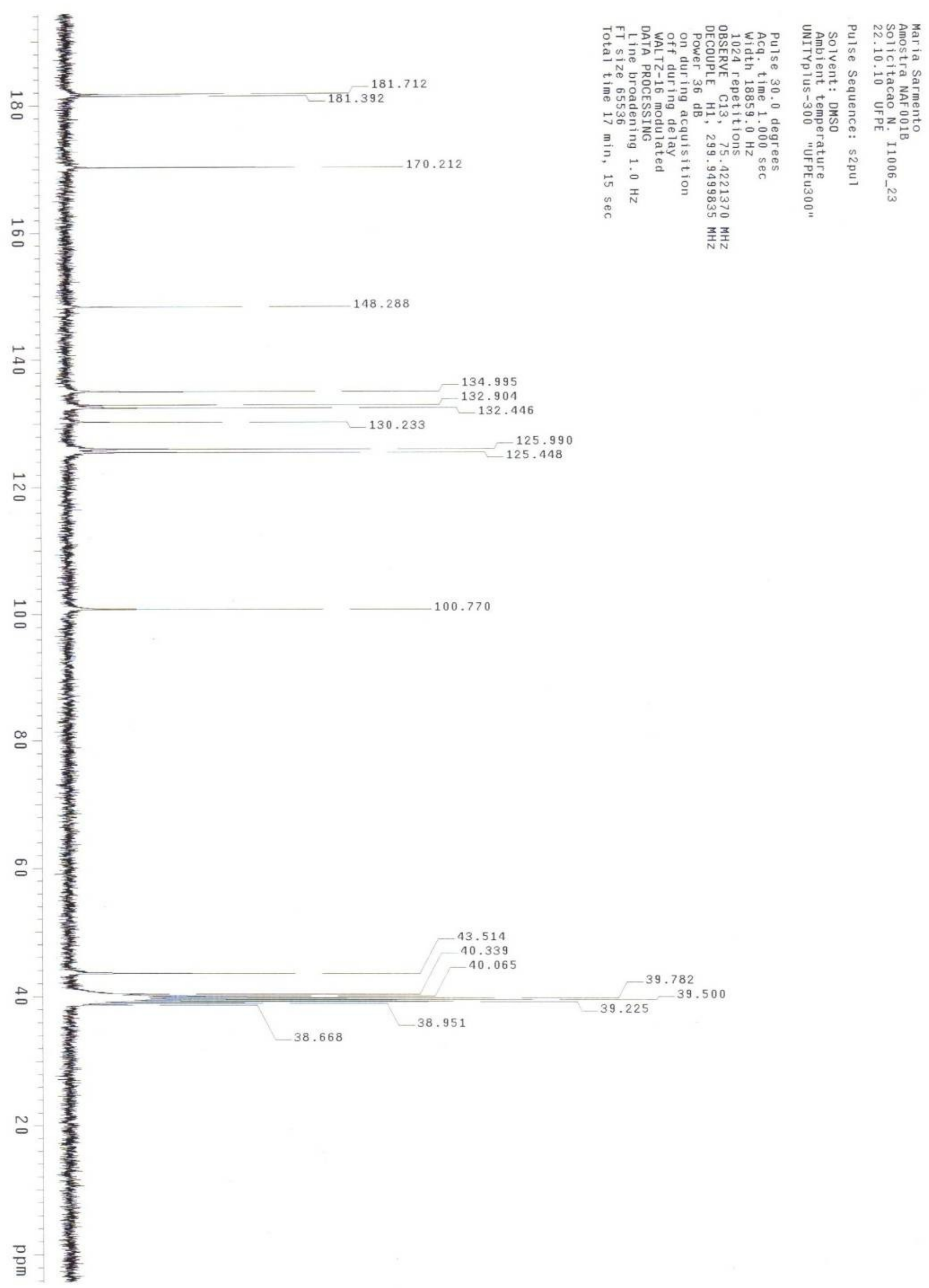

Figure S4. IR (ATR, cm^{-1}) of compound **3**.

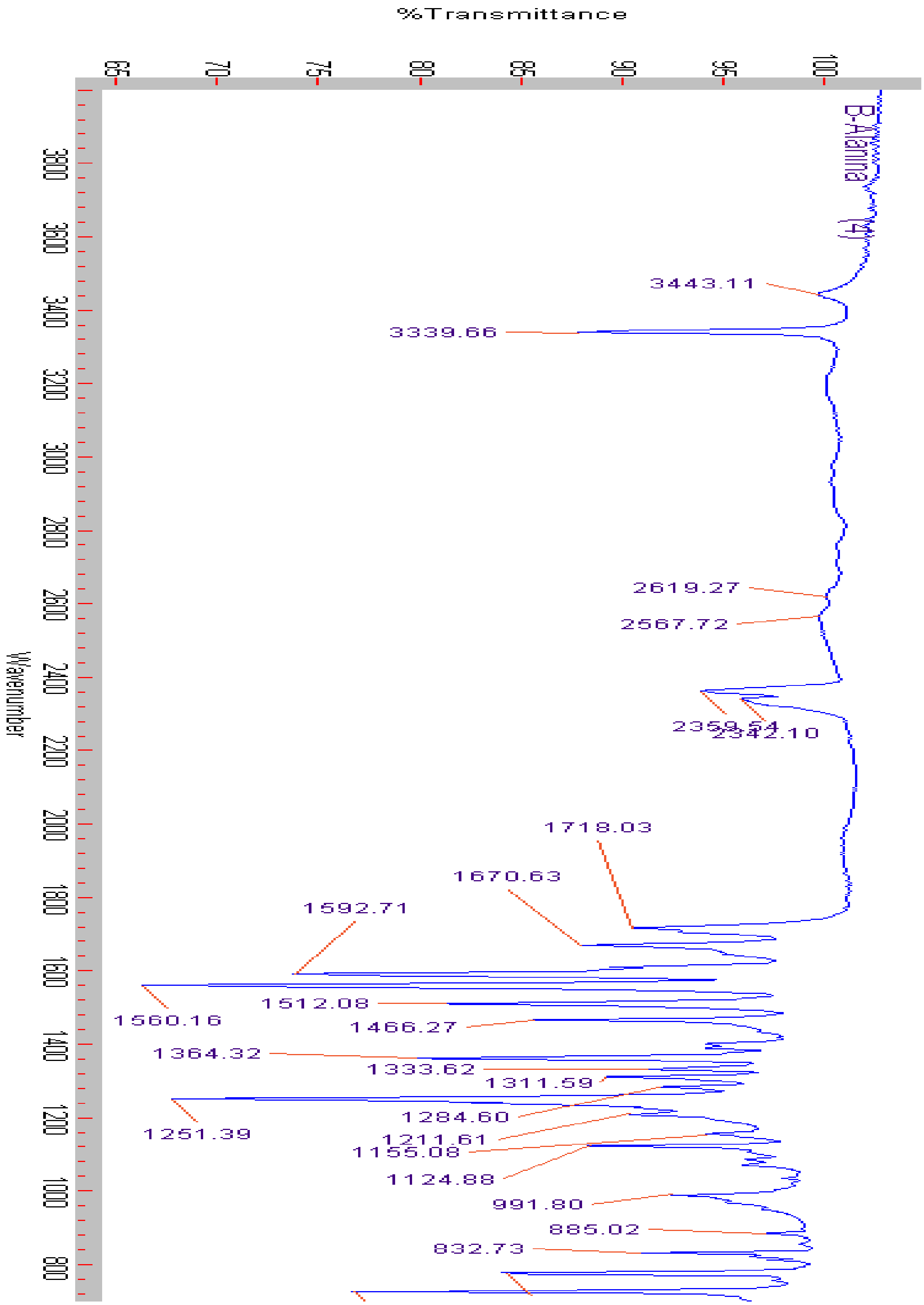

Figure S5. ^{1}H NMR (DMSO-$_{d6}$, 300 MHz) of **3**.

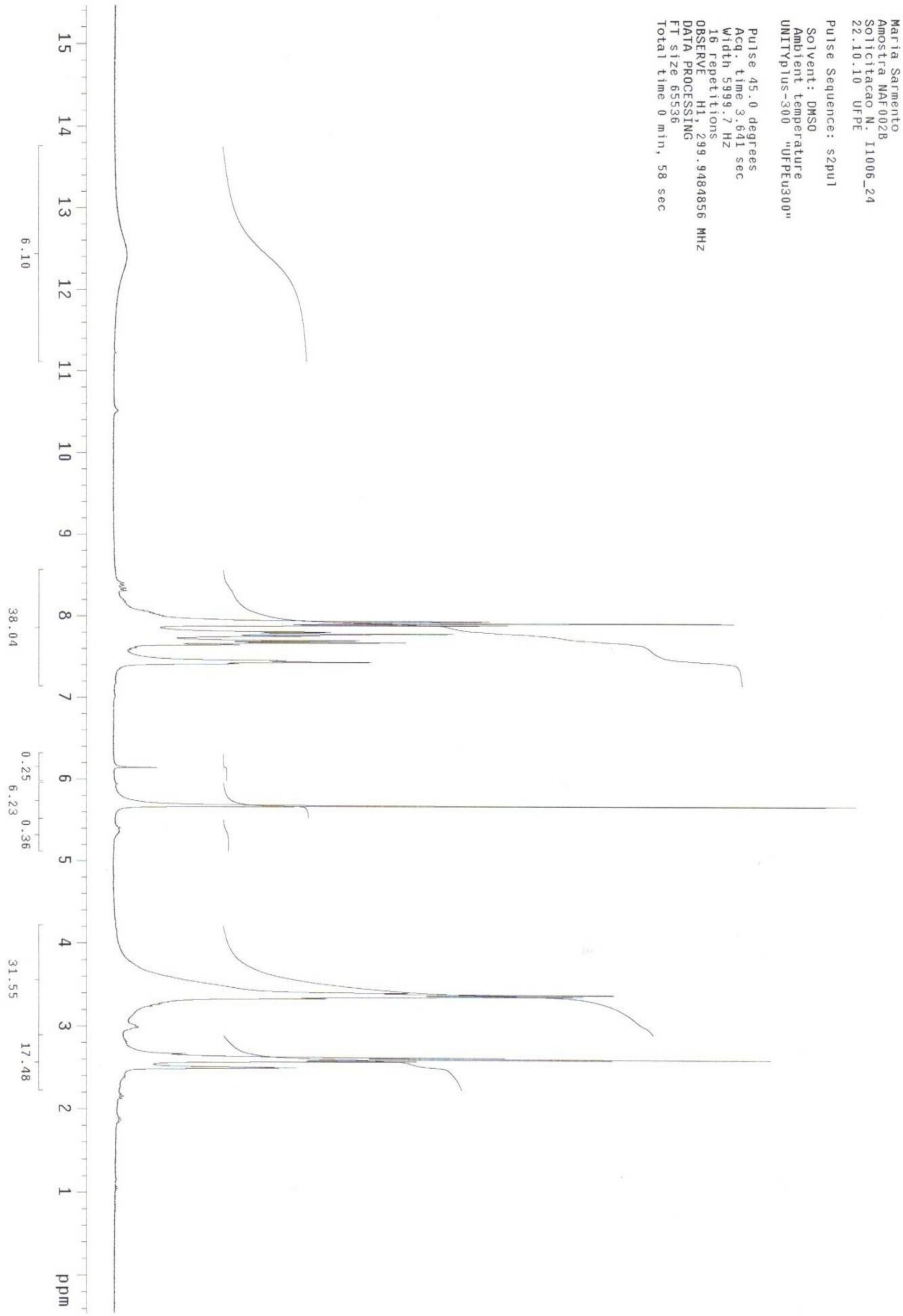

Figure S5. *Cont.*

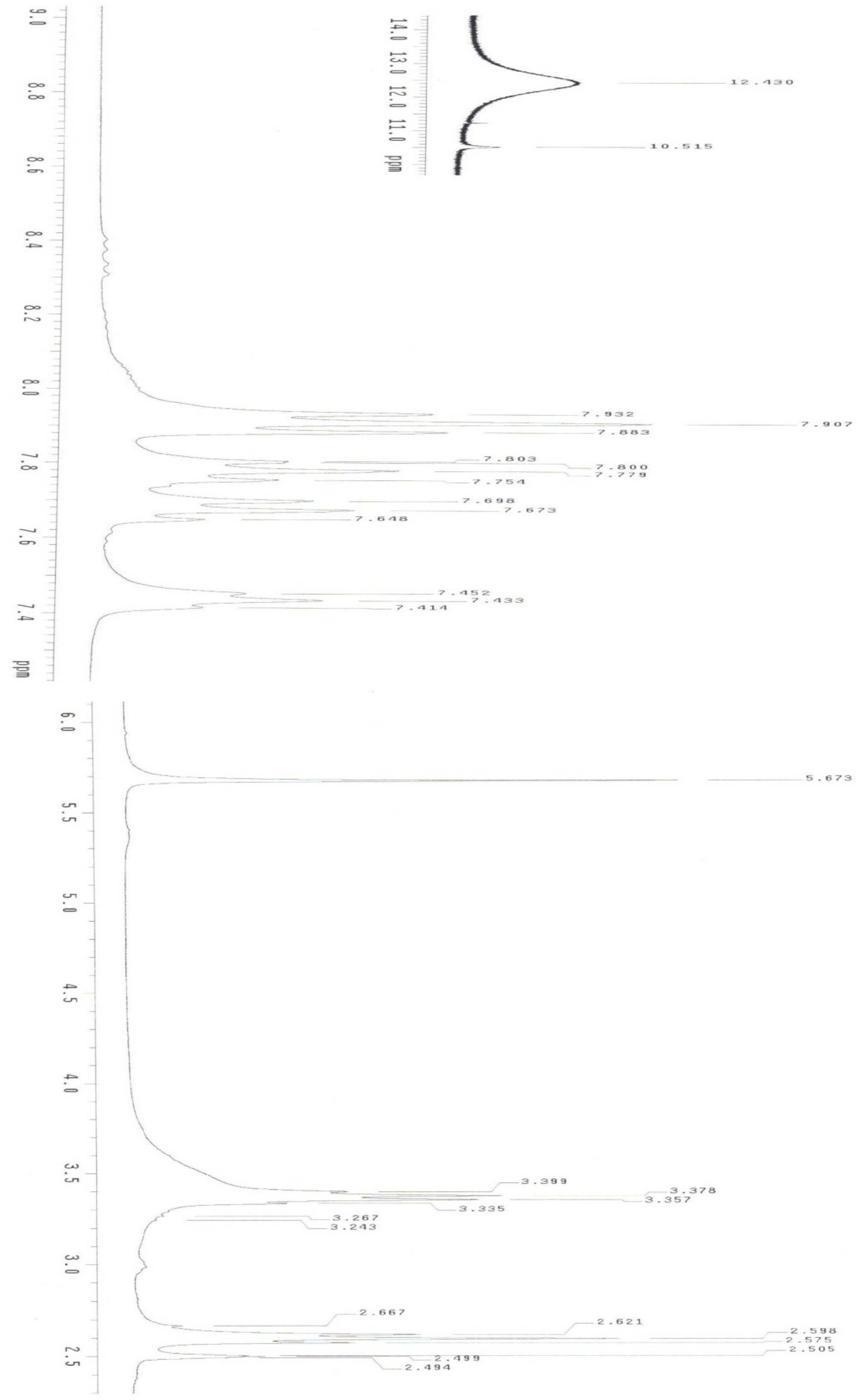

Figure S6. ^{13}C NMR (DMSO-$_{d6}$, 75 MHz) and expansion of **3**.

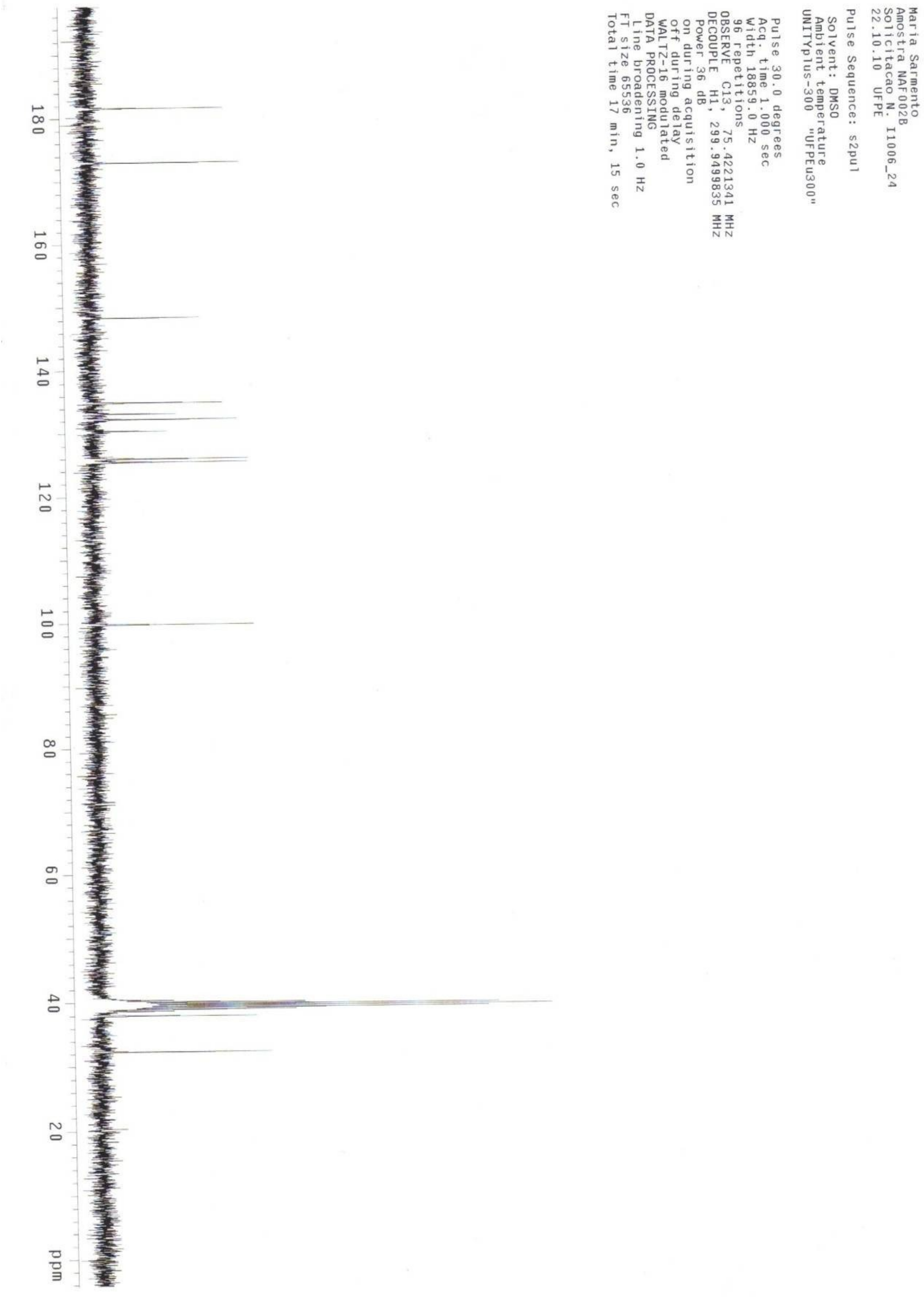

Figure S6. *Cont.*

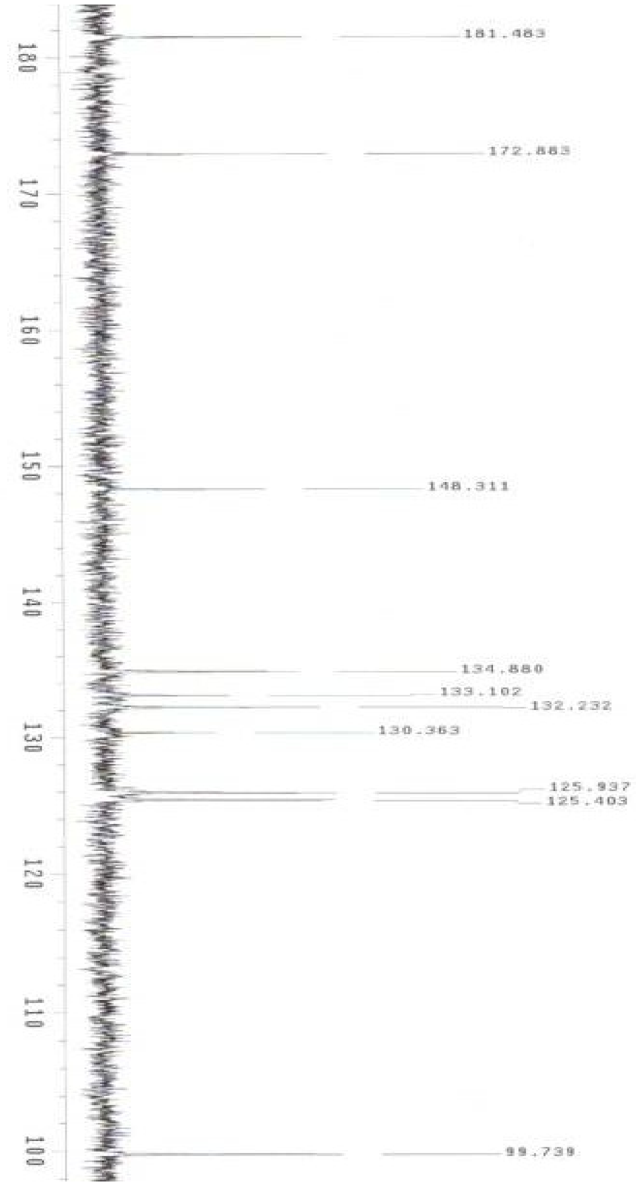

Figure S7. IR (ATR) of **4**.

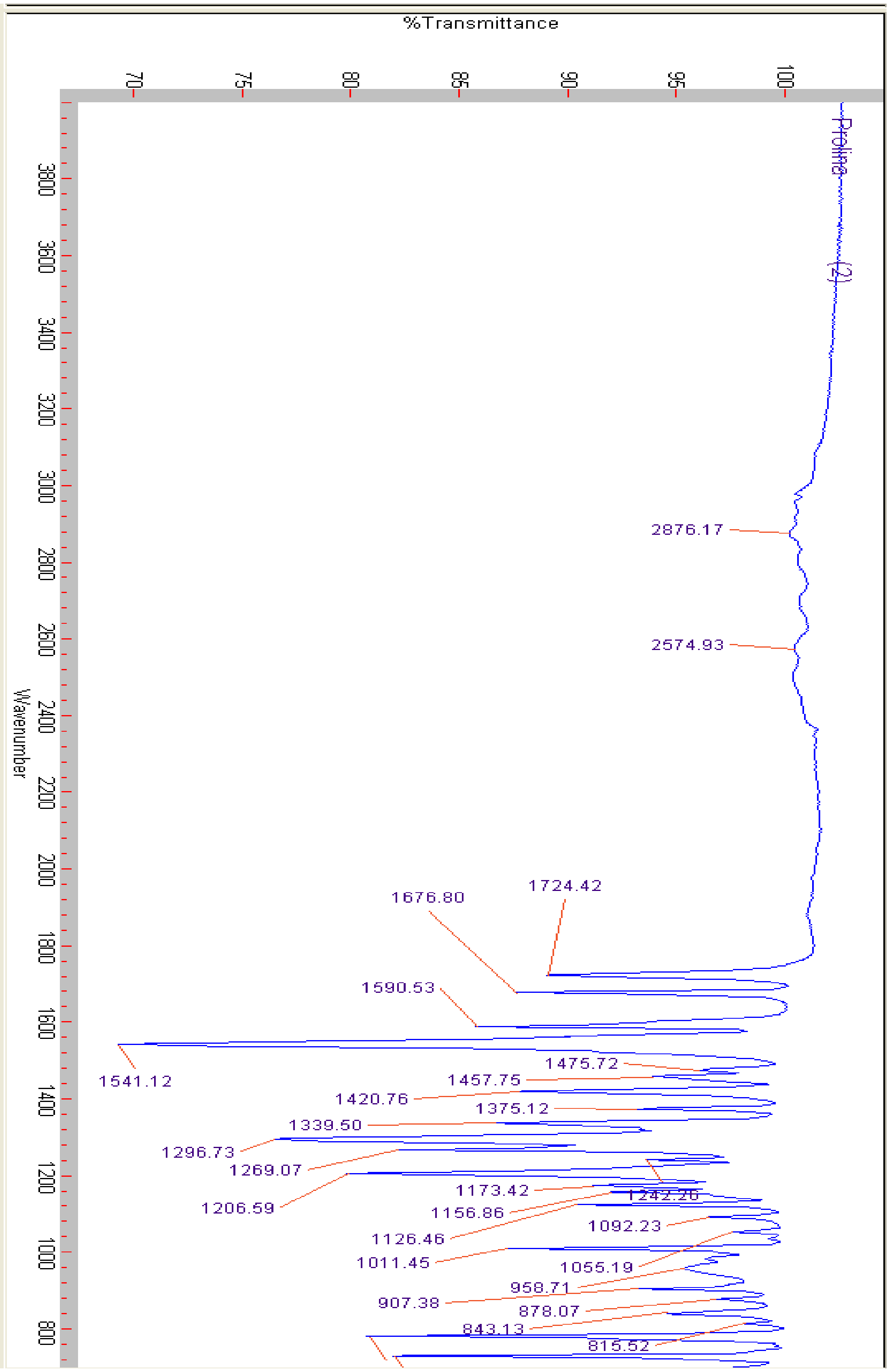

Figure S8. ^{1}H NMR (DMSO-d_6, 400 MHz) and expansions of **4**.

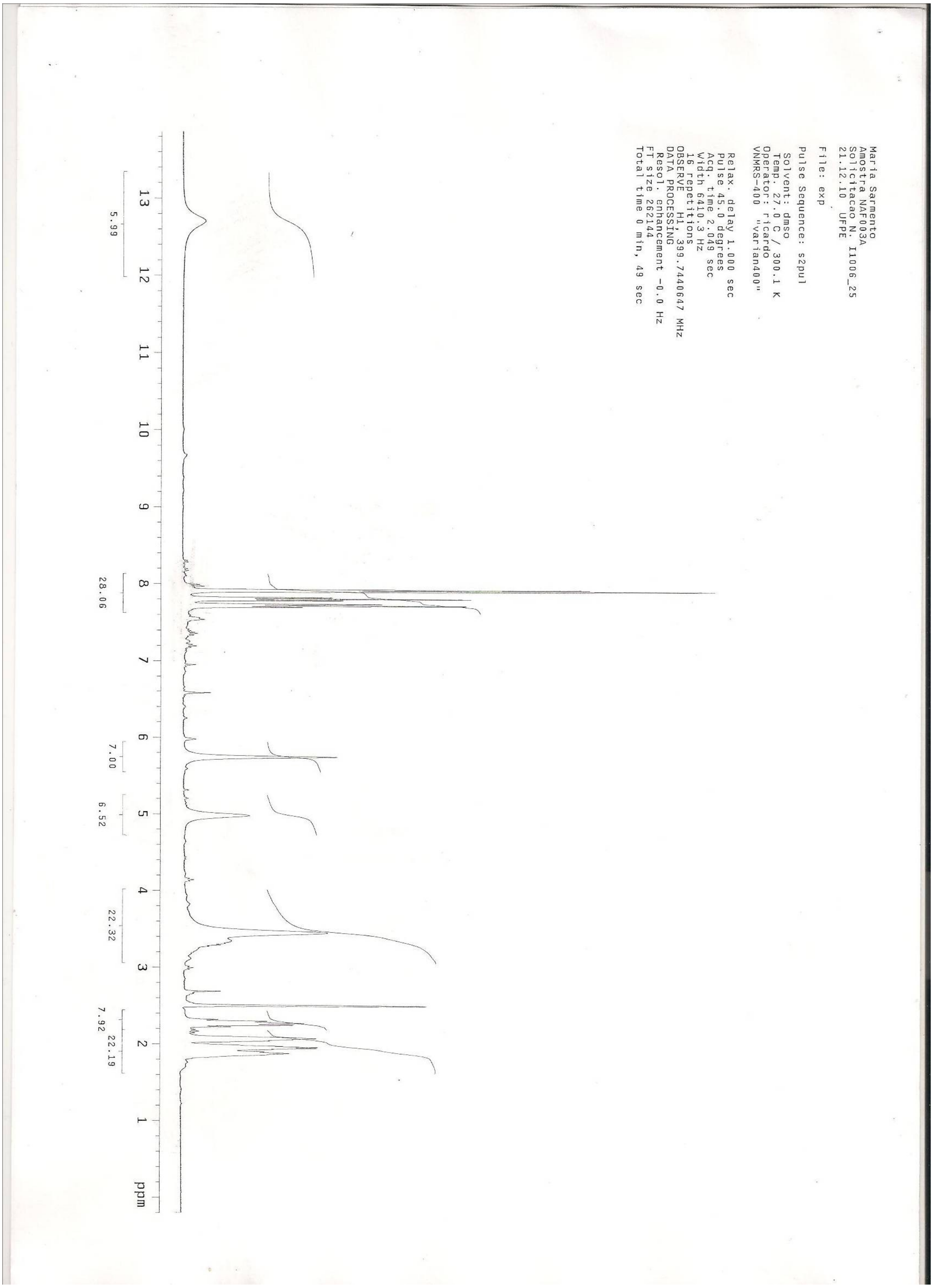

Figure S8. *Cont.*

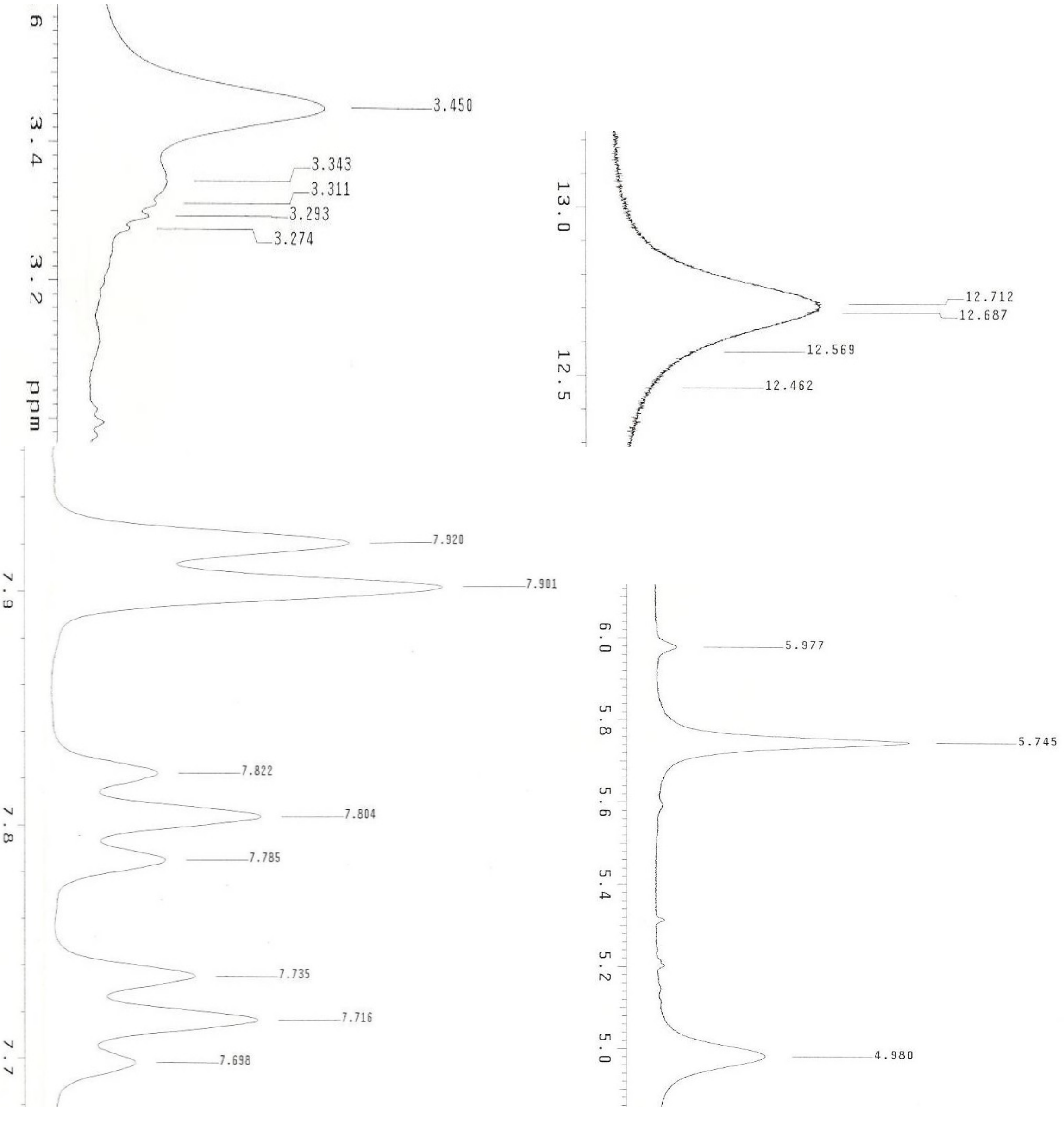

Figure S8. *Cont.*

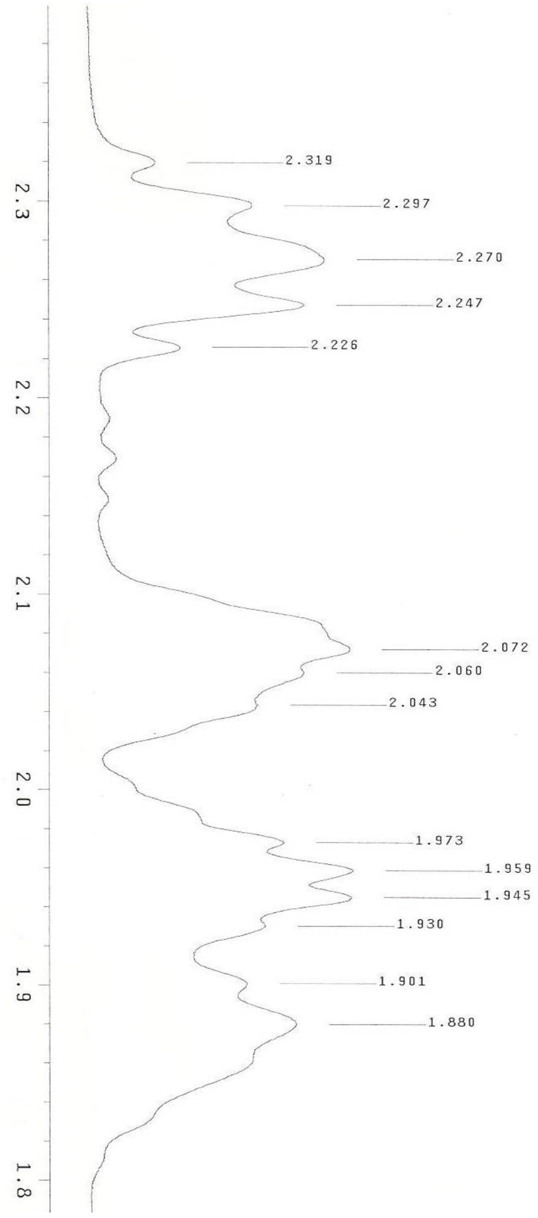

Figure S9. ^{13}C NMR (100 MHz, DMSO-$_{d6}$) and expansions of **4**.

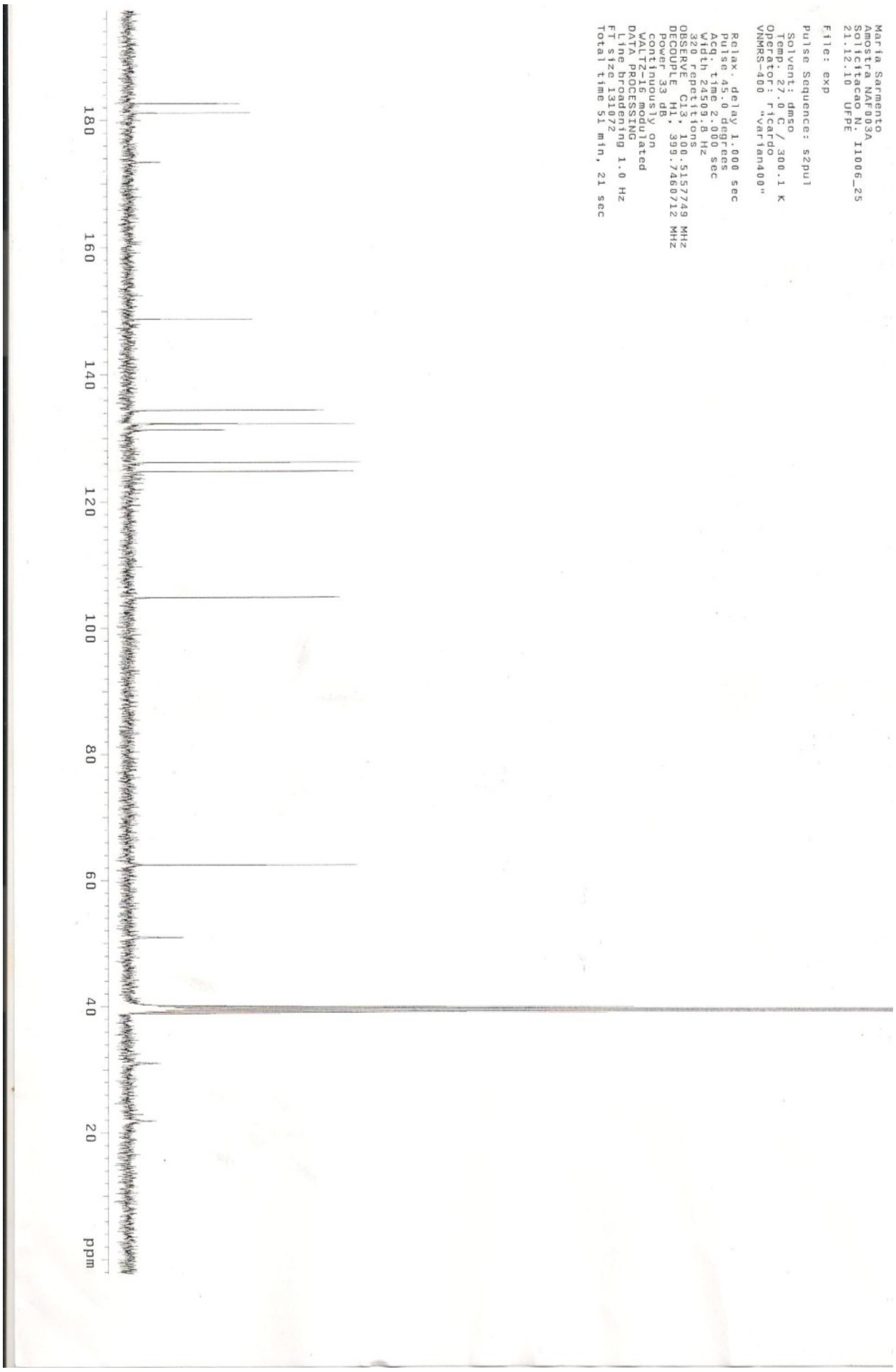

Figure S9. *Cont.*

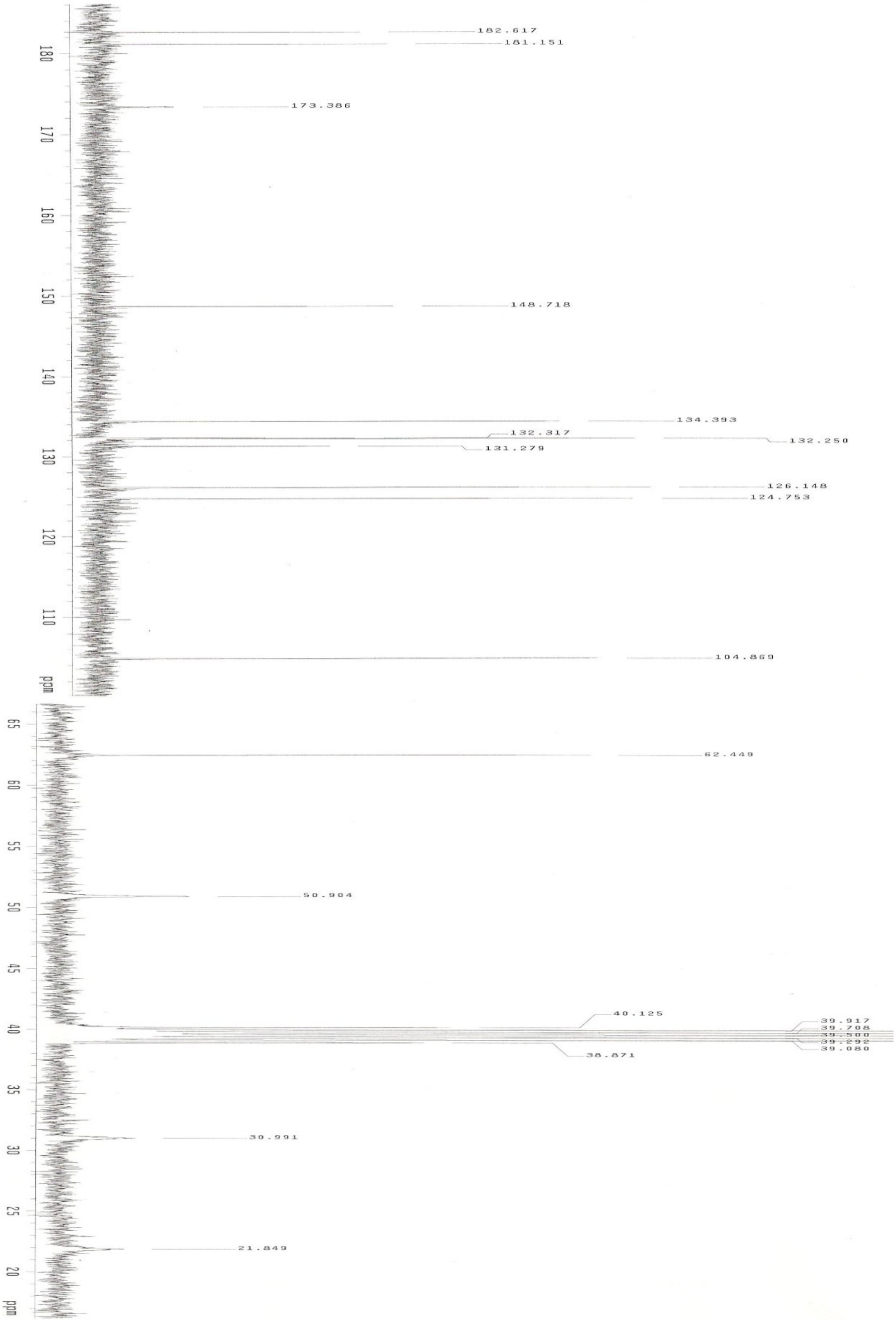

Figure S10. IR (ATR) of **5**.

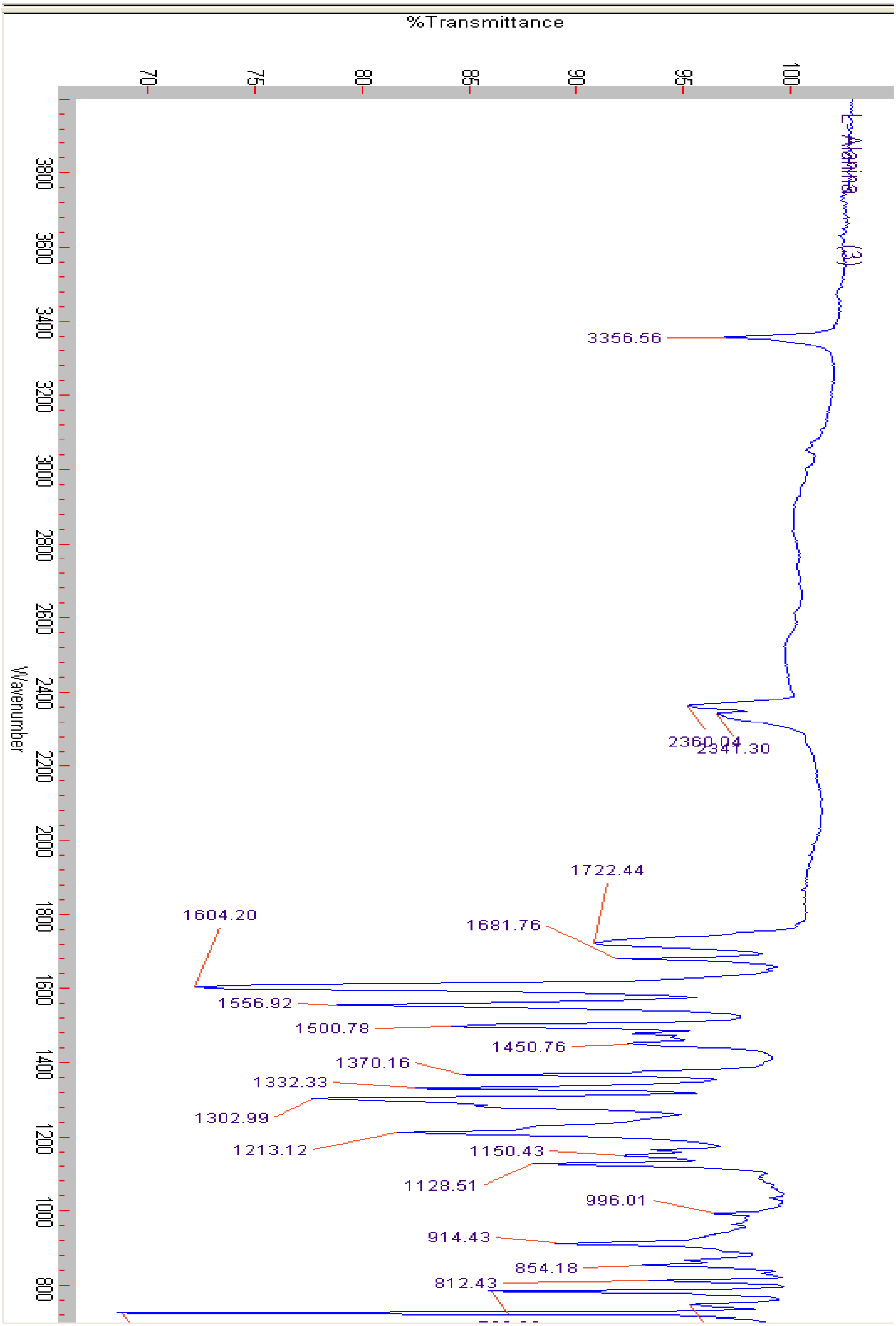

Figure S11. ^{1}H NMR (300 MHZ, DMSO-$_{d6}$) and expansion of **5**.

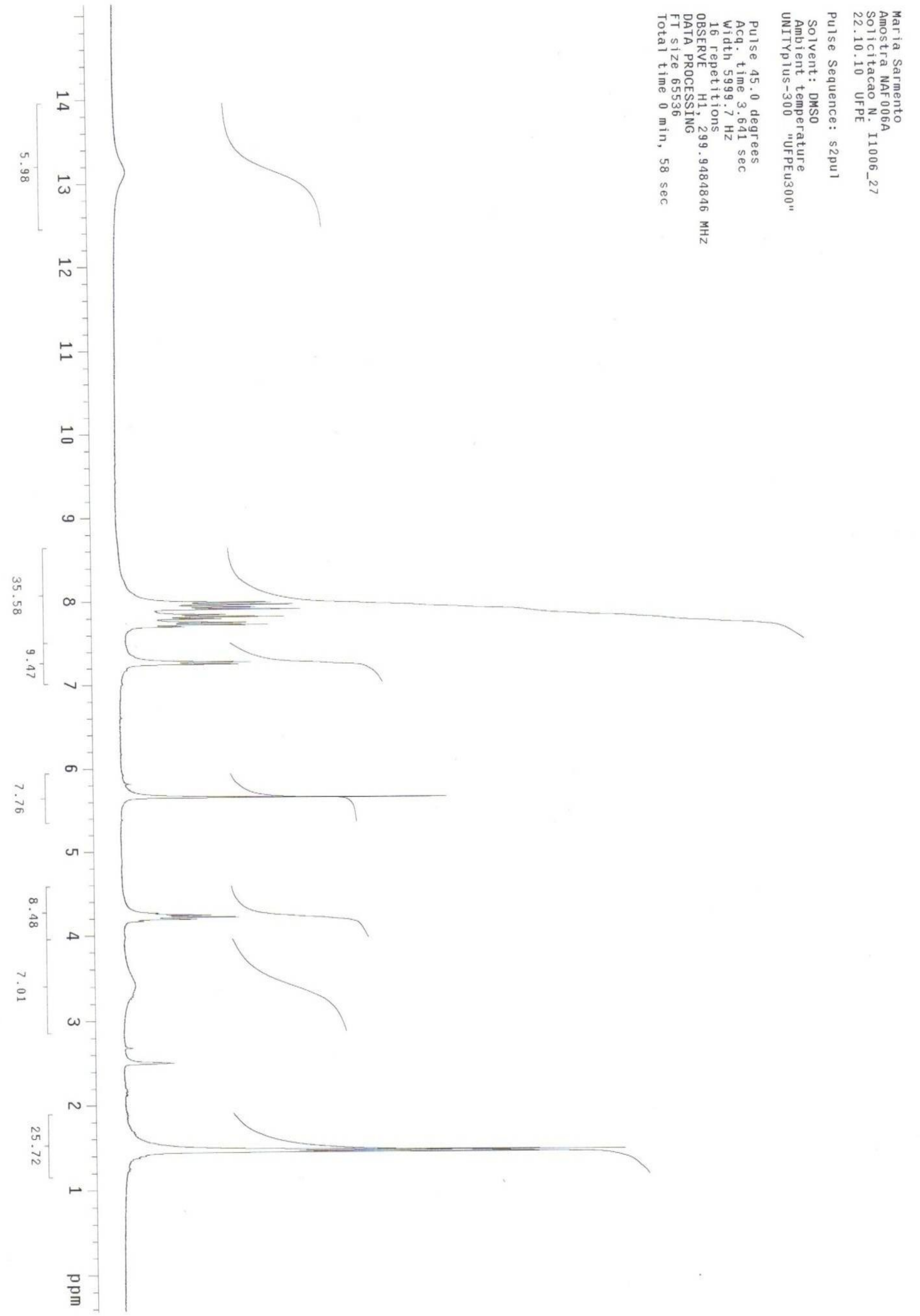

Figure S11. *Cont.*

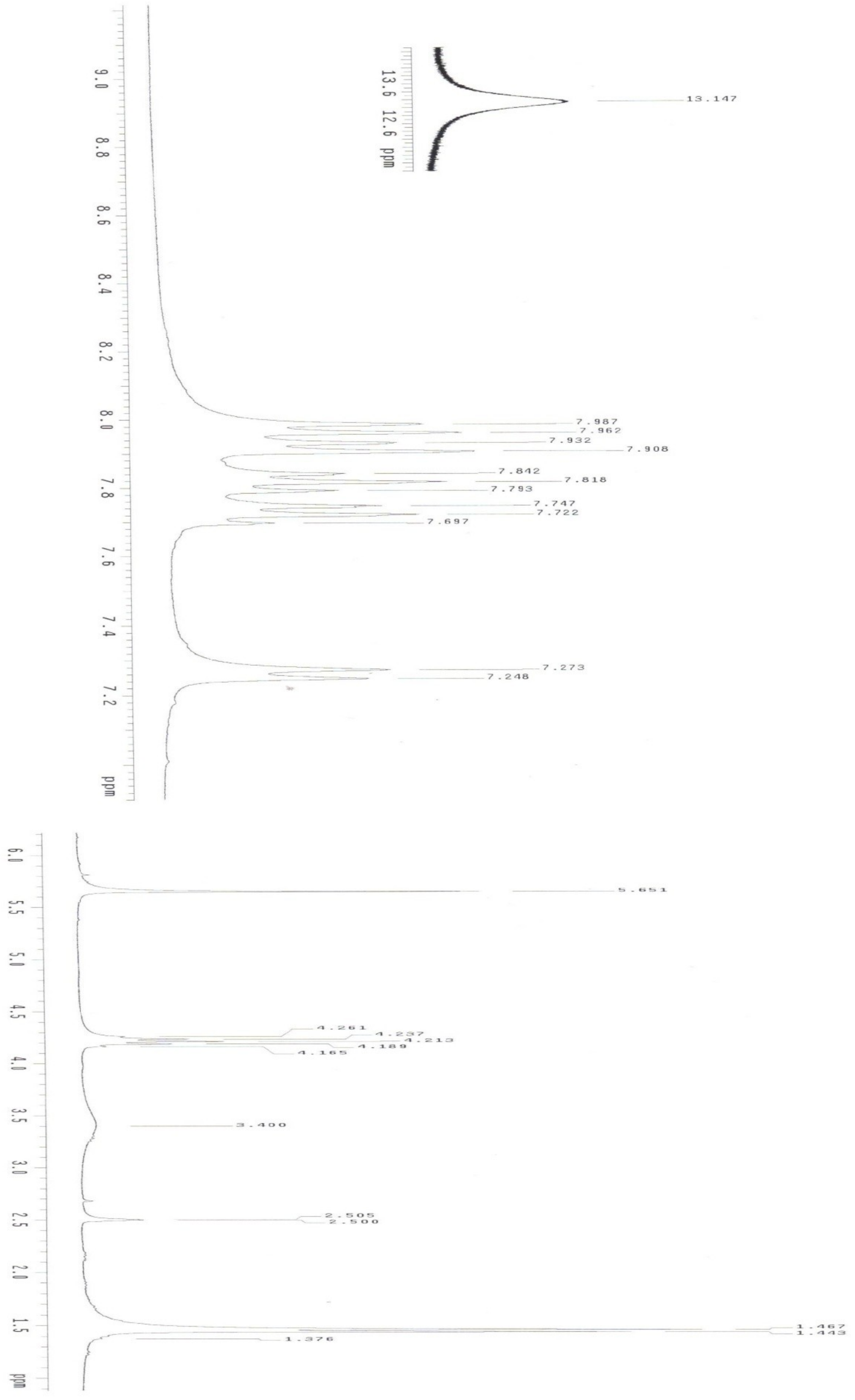

Figure S12. ^{13}C NMR (75 MHZ, DMSO-$_{d6}$) and expansions of **5**.

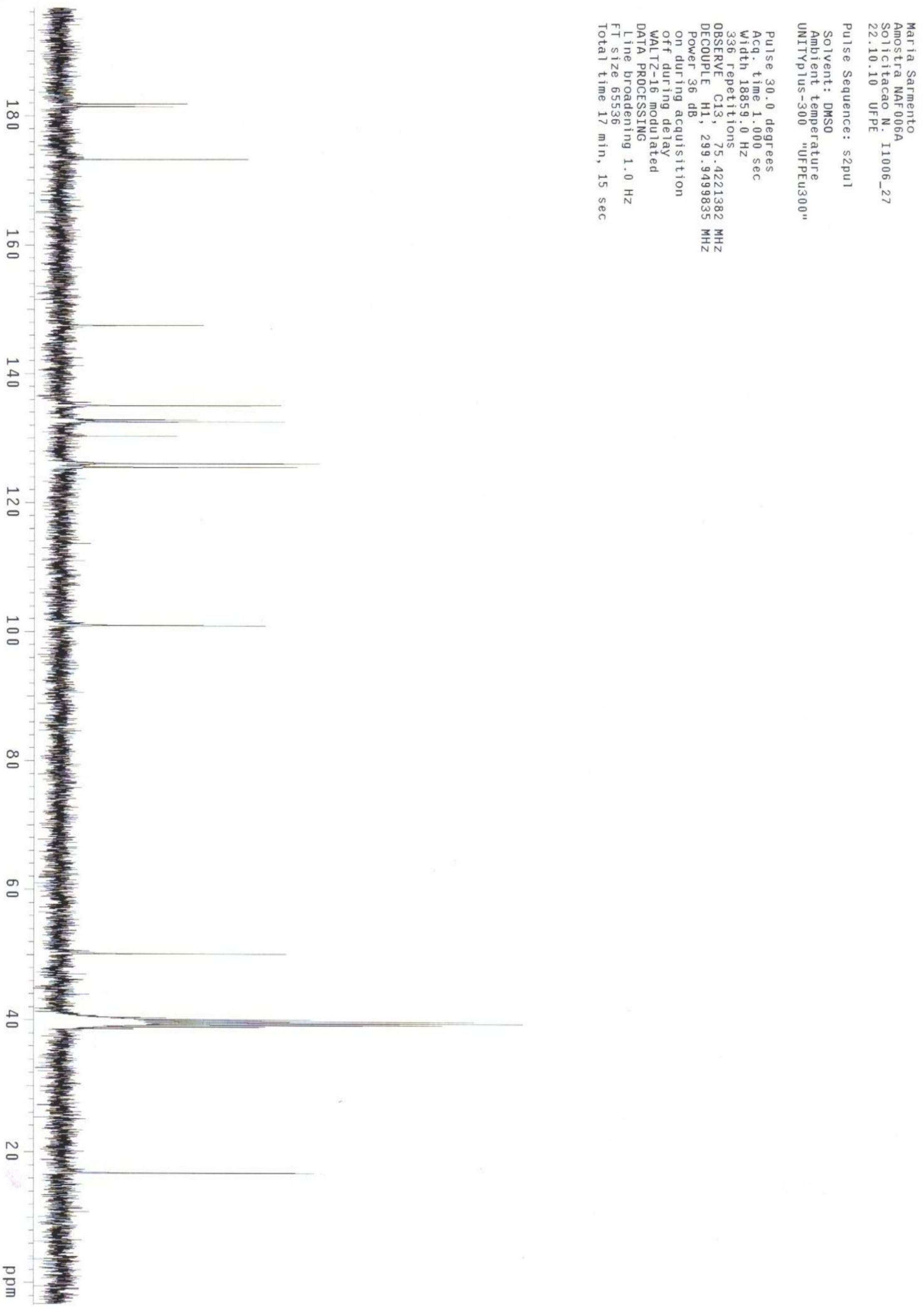

Figure S12. *Cont.*

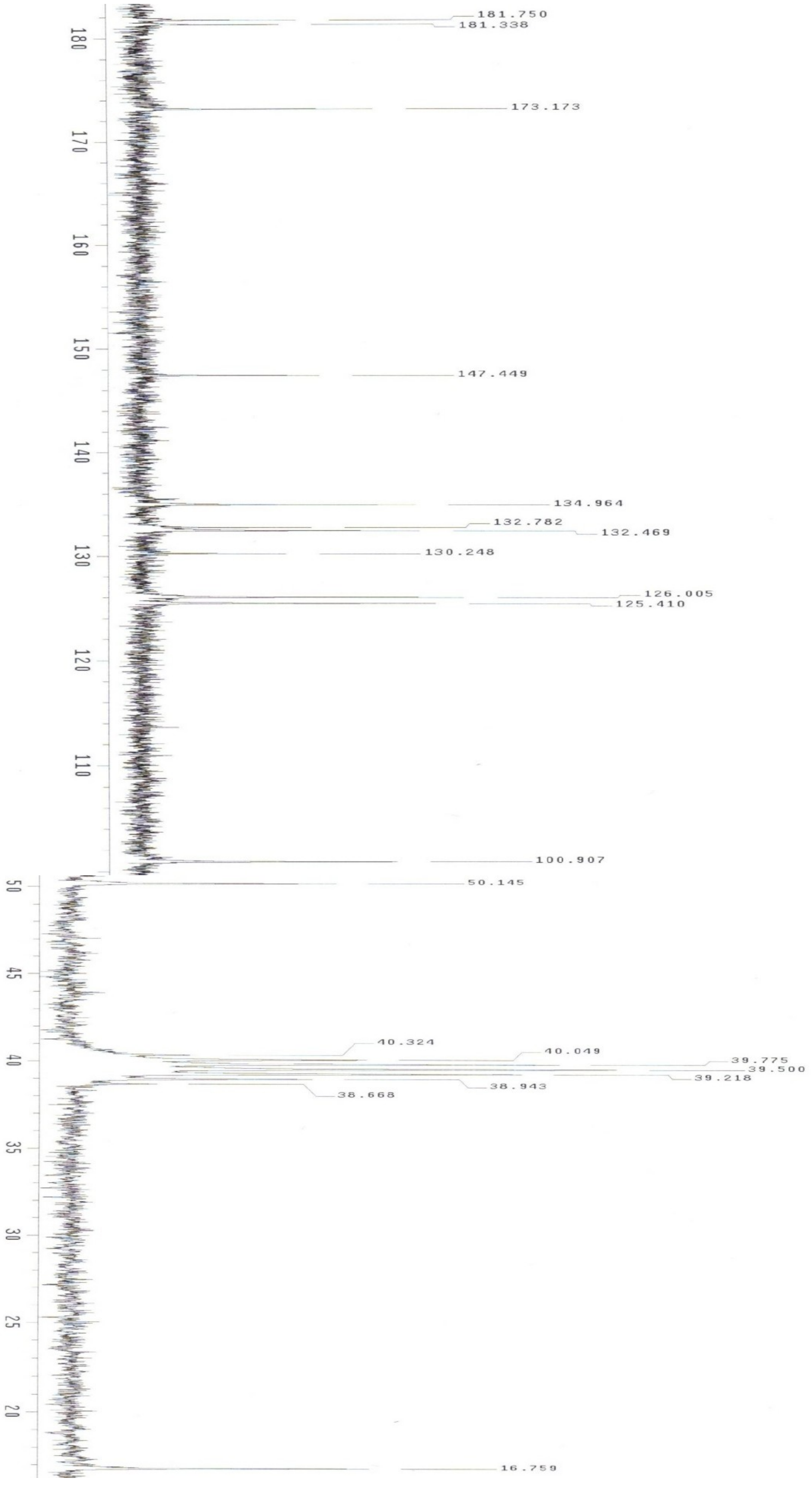

Figure S13. IR (KBr, cm^{-1}) of **6**.

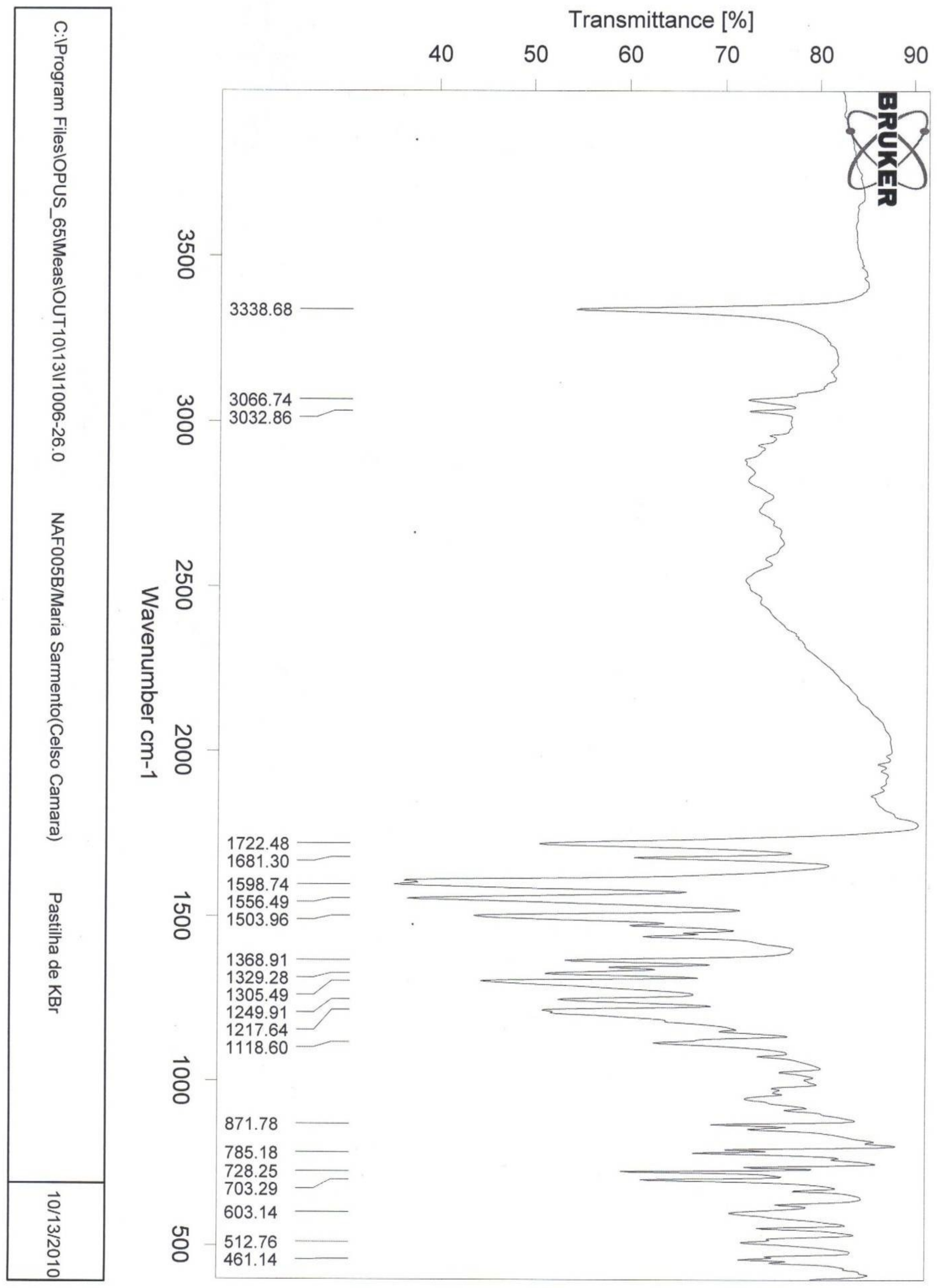

Figure S14. ^{1}H NMR (400 MHz, DMSO-$_{d6}$) and expansion of **6**.

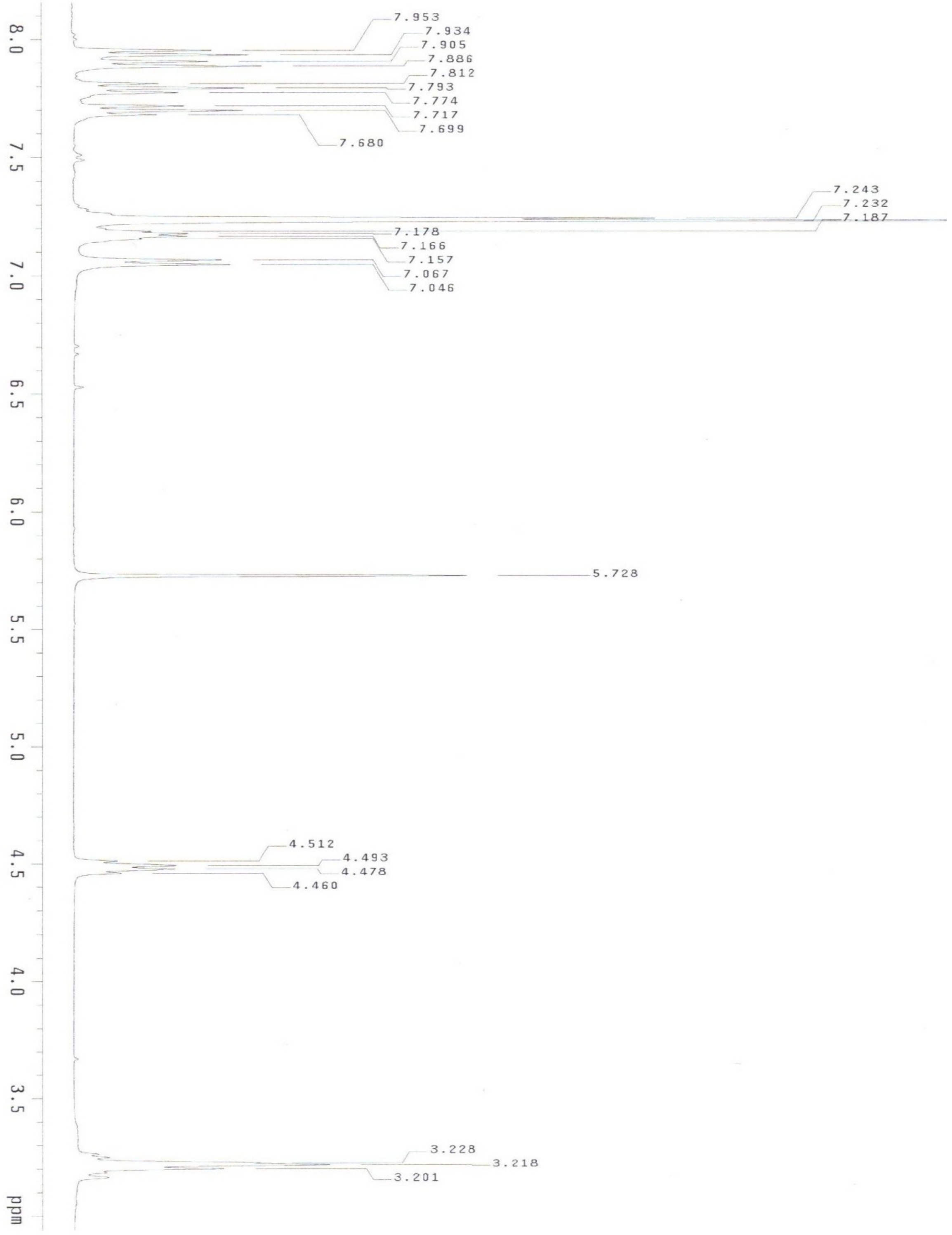

Figure S14. *Cont.*

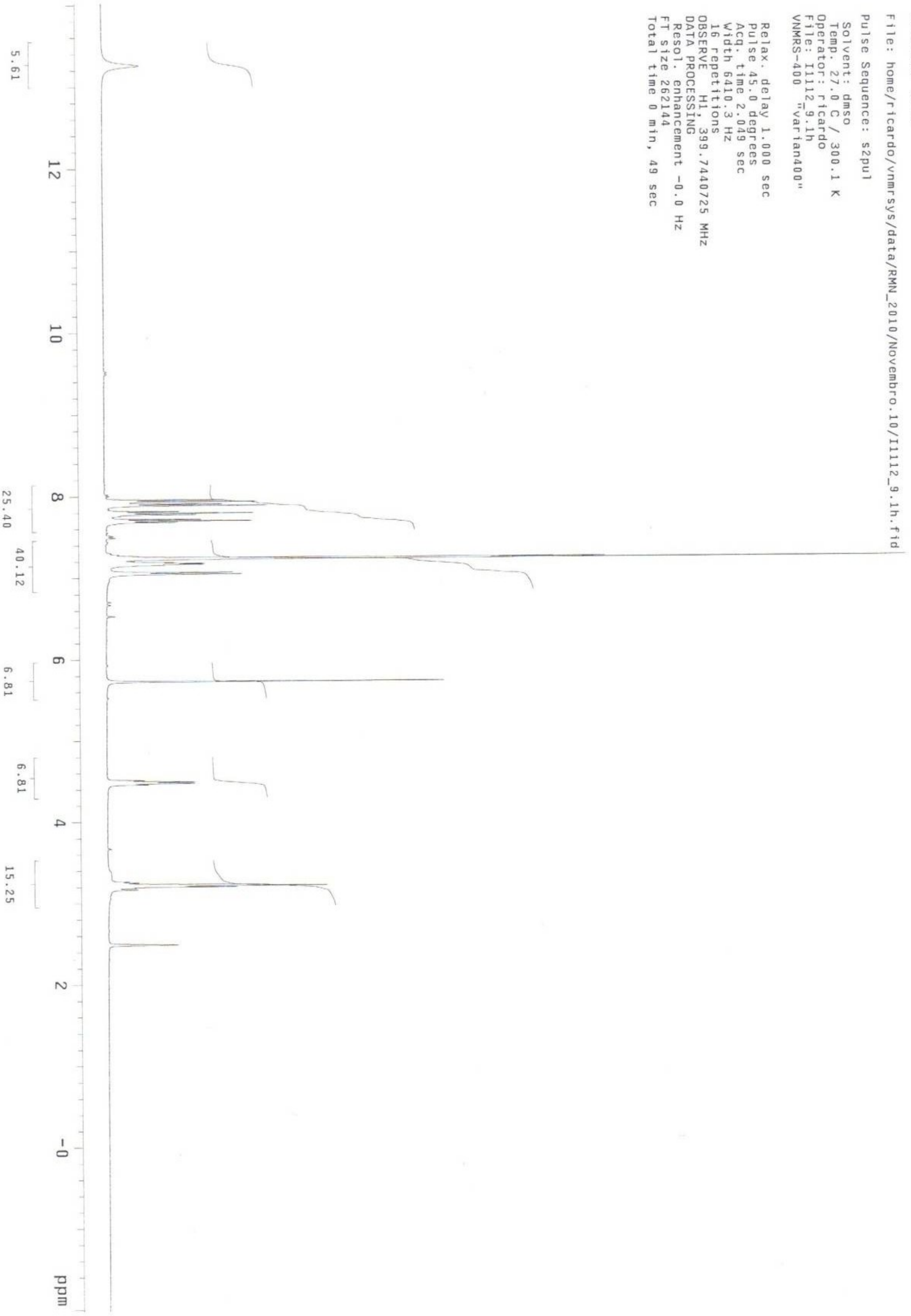

Figure S15. ^{13}C NMR (100 MHz, DMSO-$_{d6}$) of **6**.

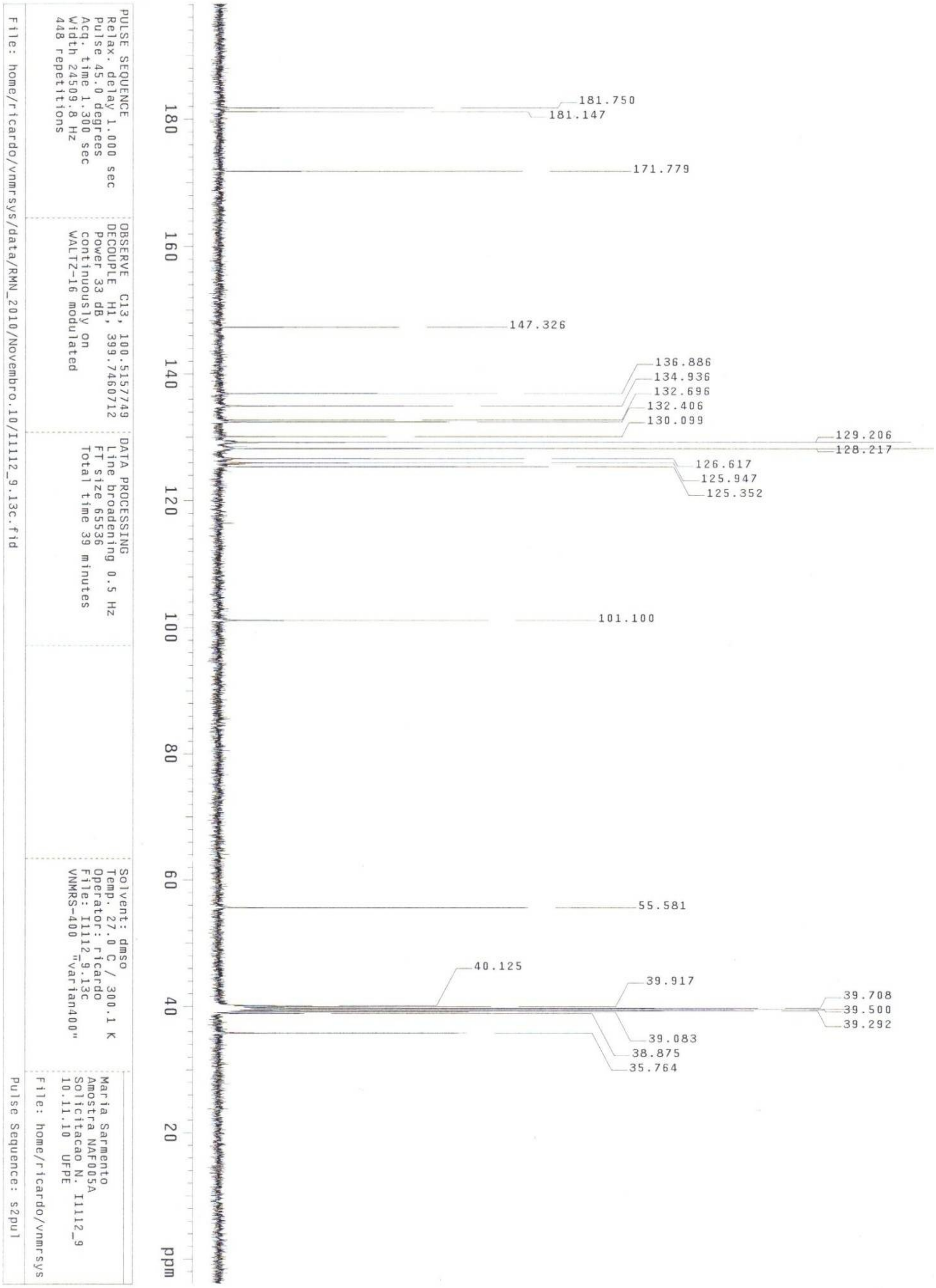

Figure S16. IR (ATR) of **7**.

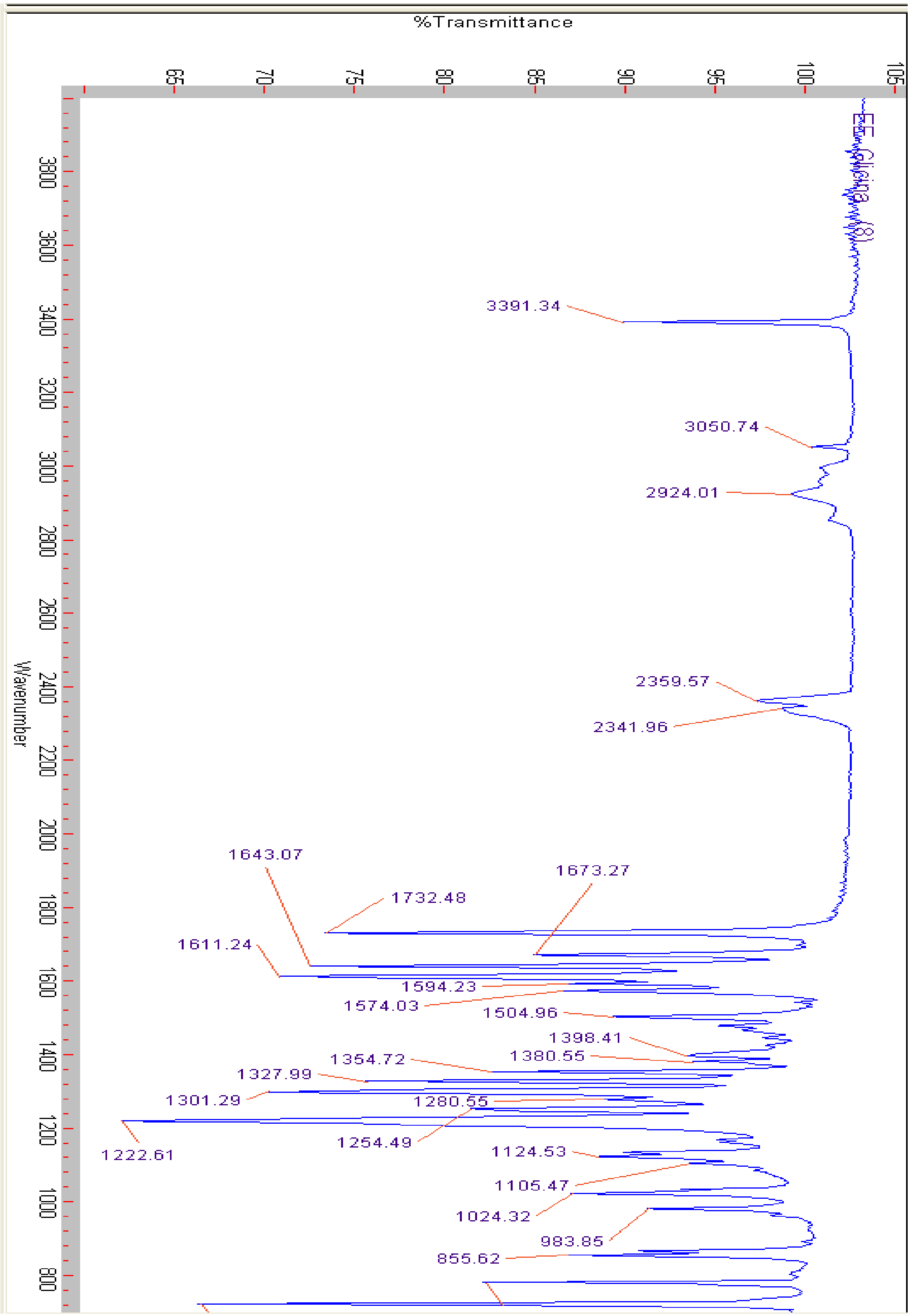

Figure S17. ^{1}H NMR (200 MHz, $CDCl_3$) and expansion of **7**.

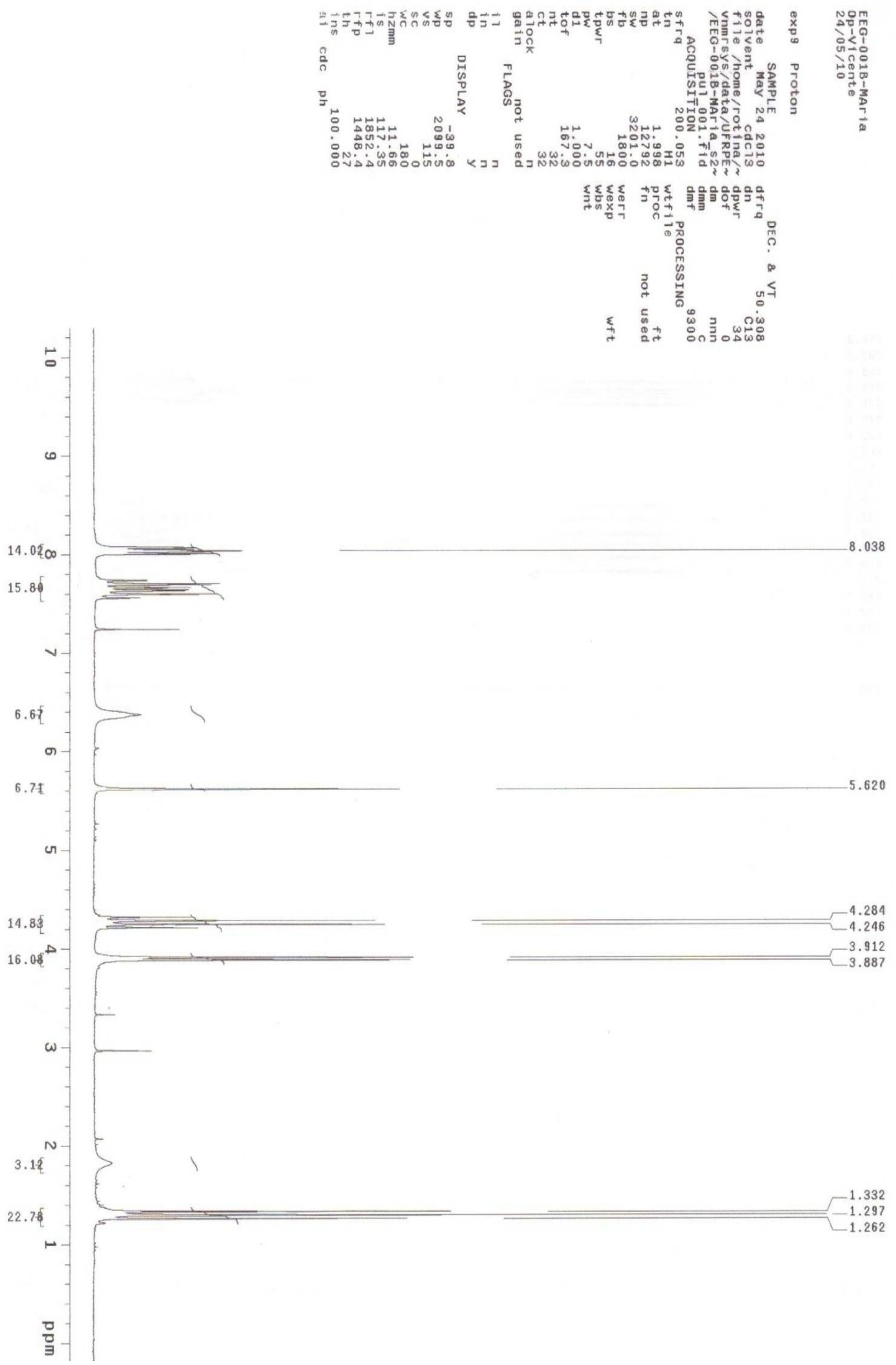

Figure S17. *Cont.*

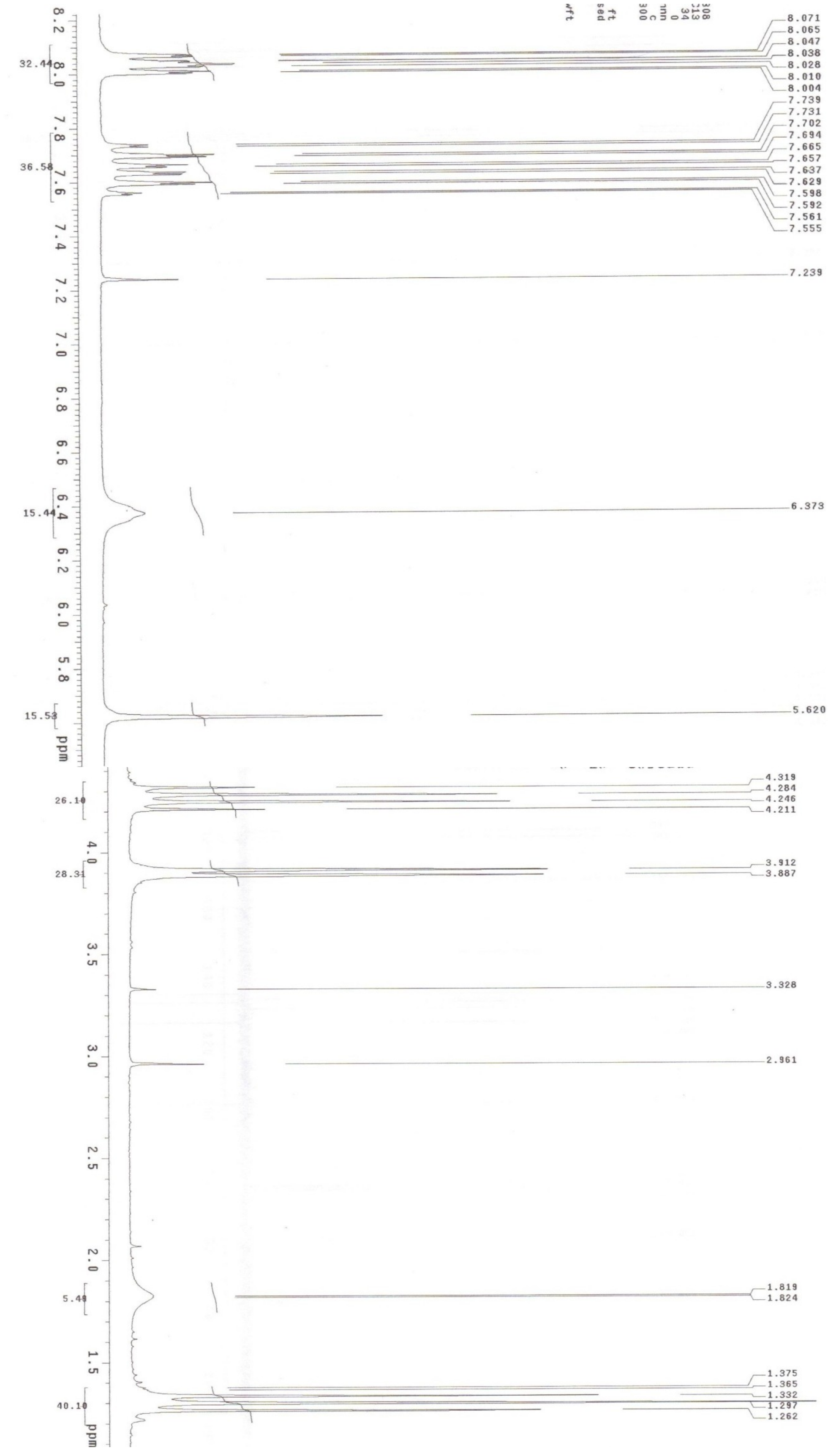

Figure S18. ^{13}C NMR (APT, 50 MHz, $CDCl_3$) of **7**.

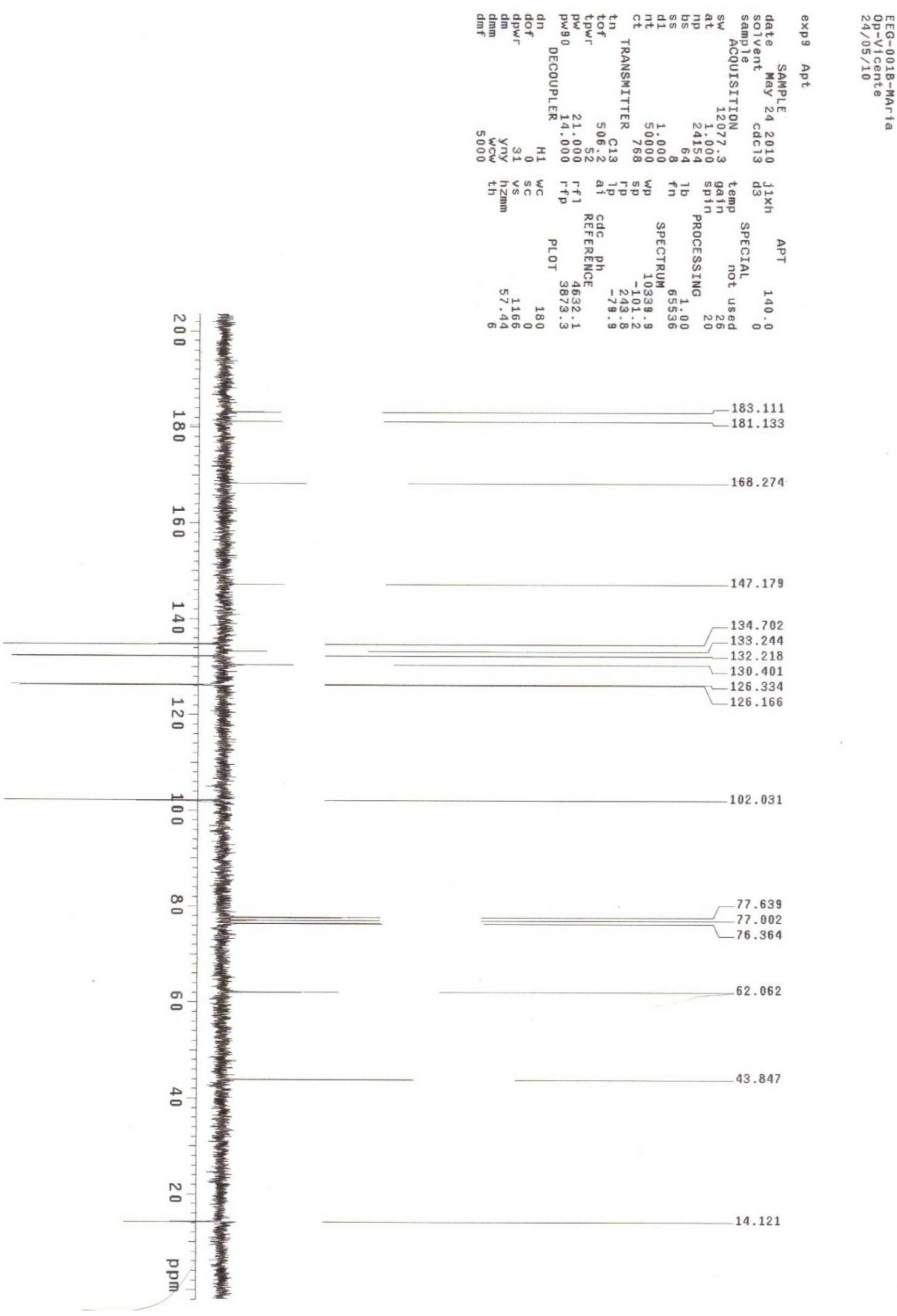

Figure S19. IR (ATR, cm^{-1}) of 9.

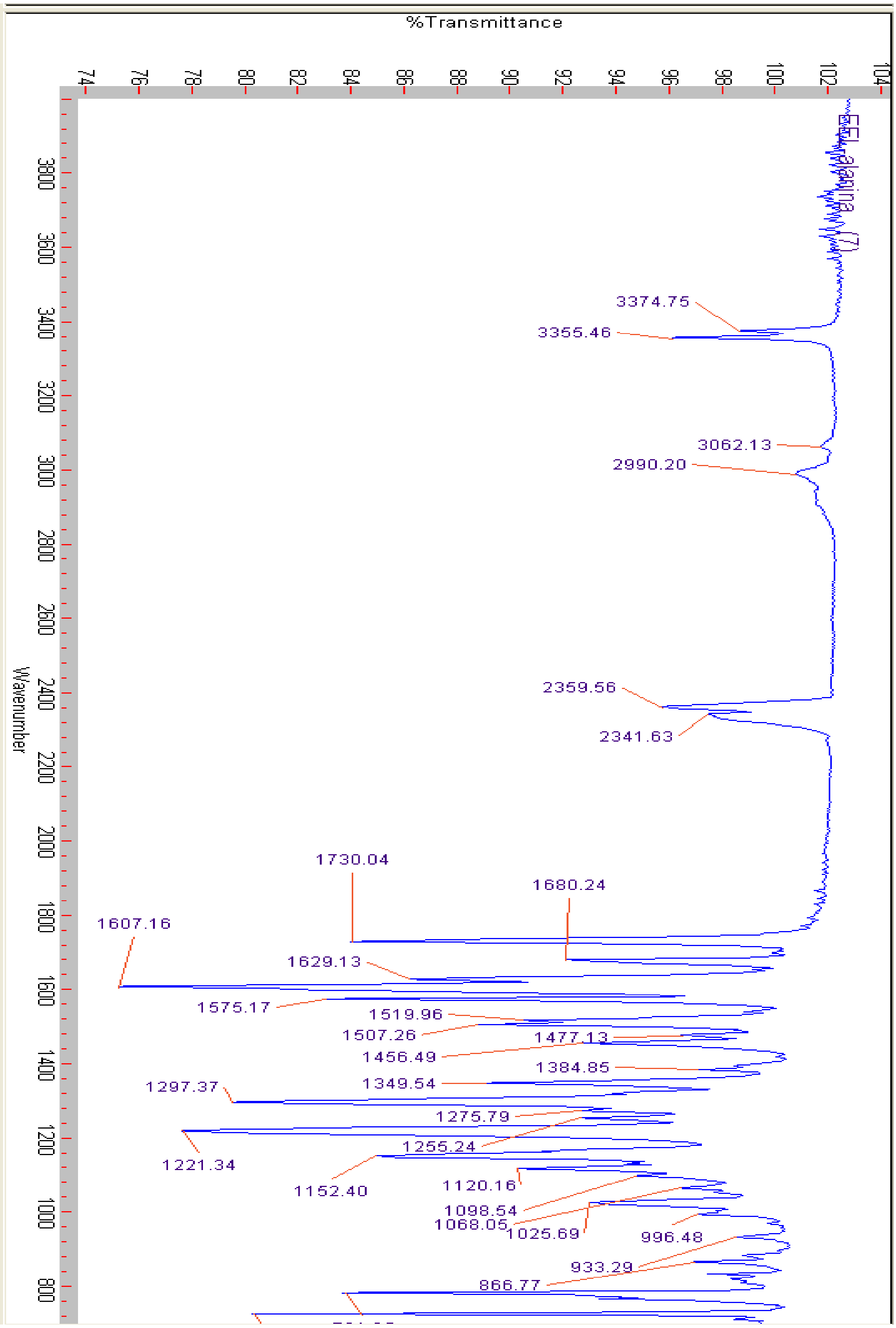

Figure S20. ^{1}H NMR (200 MHz, $CDCl_3$) and expansion of **9**.

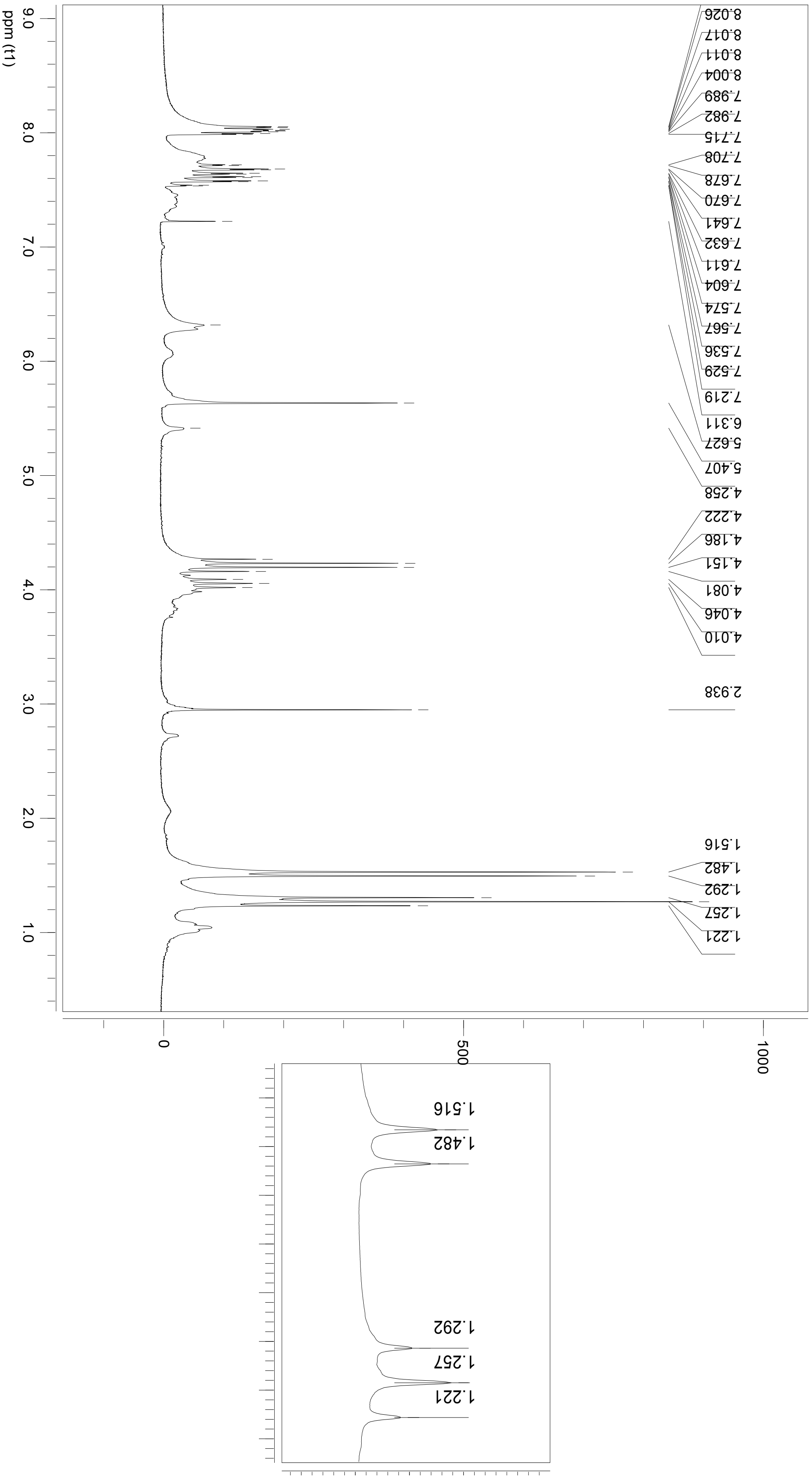

Figure S21. ^{13}C NMR-APT (δ, $CDCl_3$, 50 MHz) of **9**.

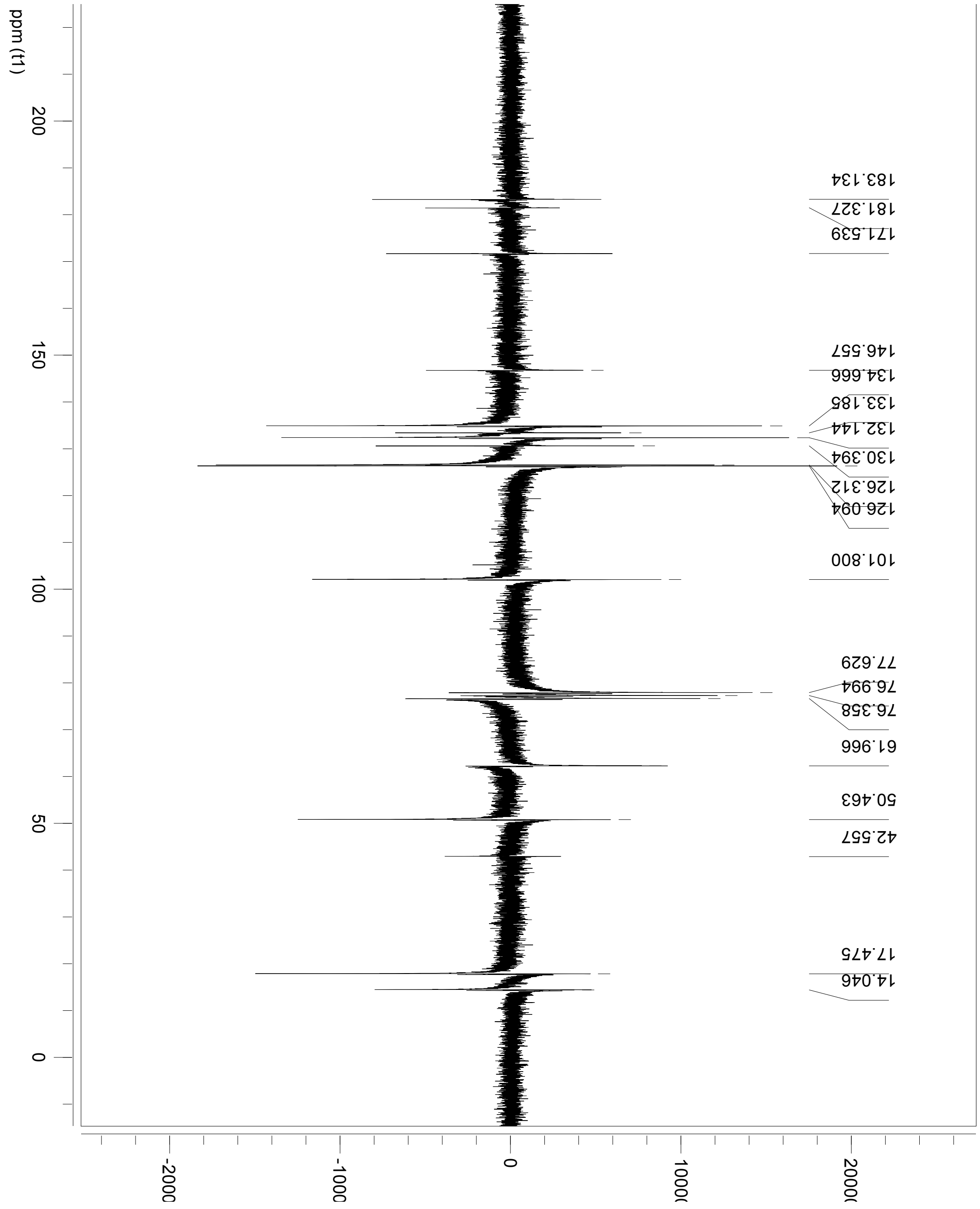

Figure S22. IR (ATR) of **8**.

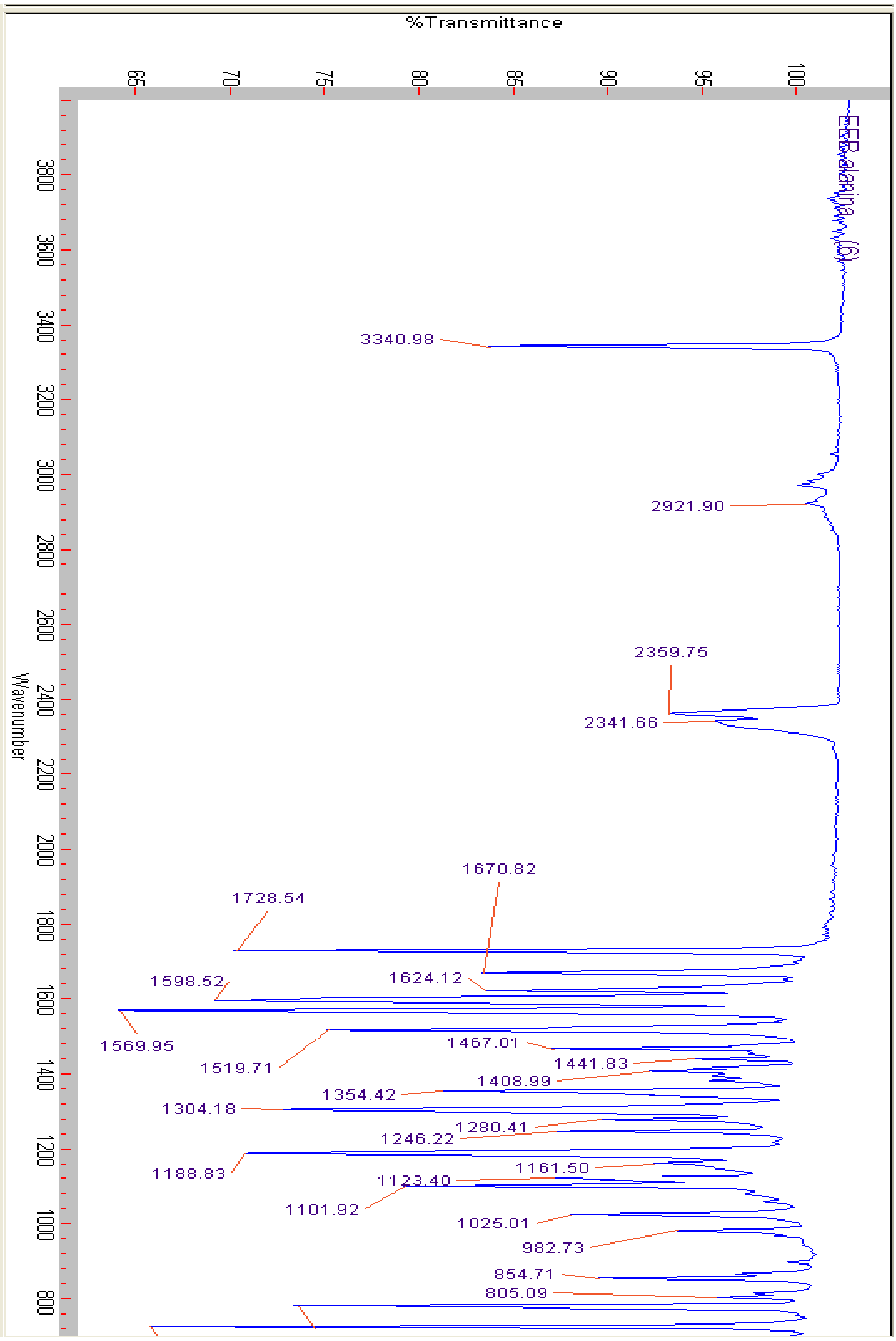

Figure S23. ^{1}H NMR (δ, $CDCl_3$, 200 MHz) of 8.

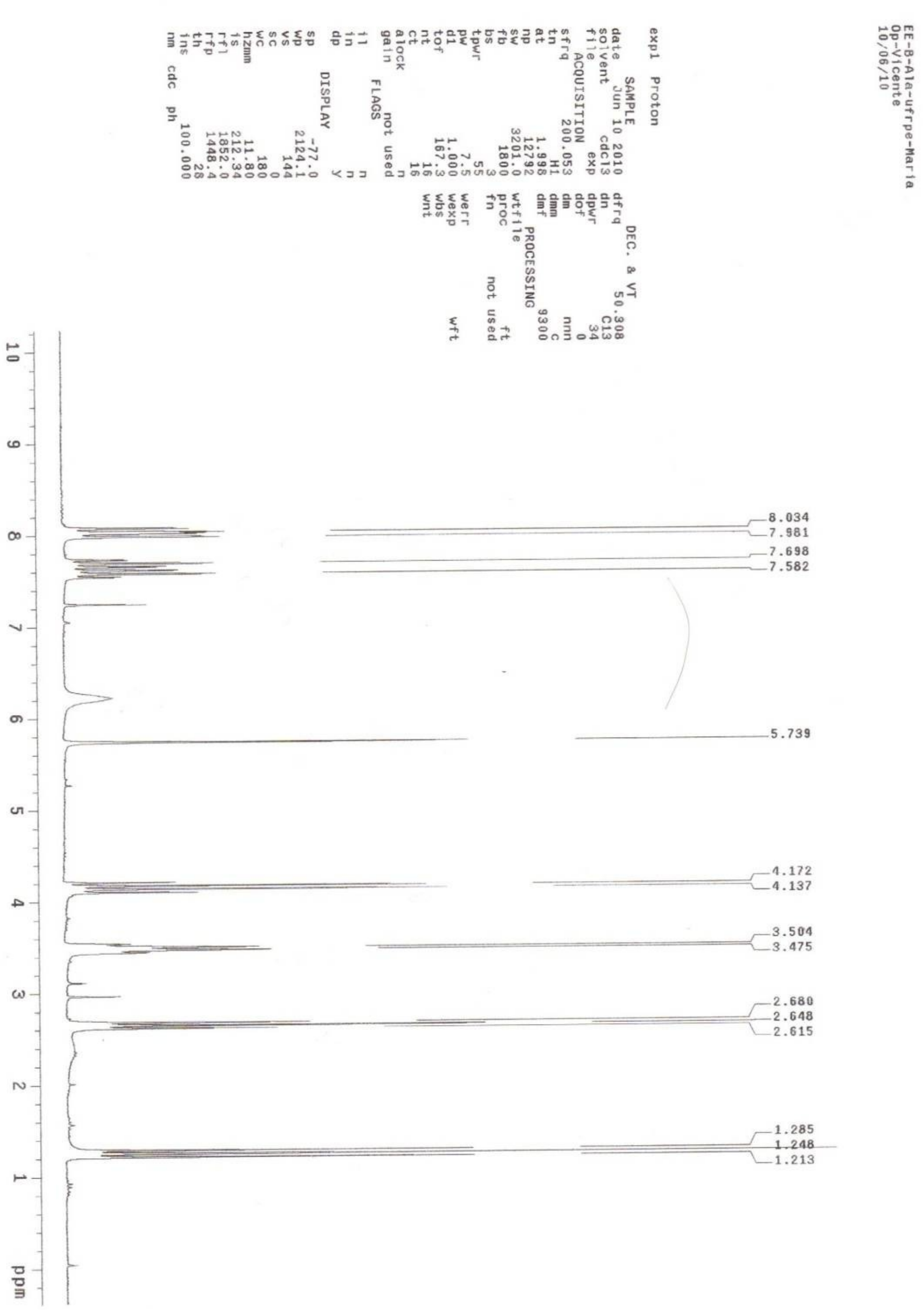

Figure S24. ^{1}H NMR (δ, $CDCl_3$, 200 MHz) expansions of 8

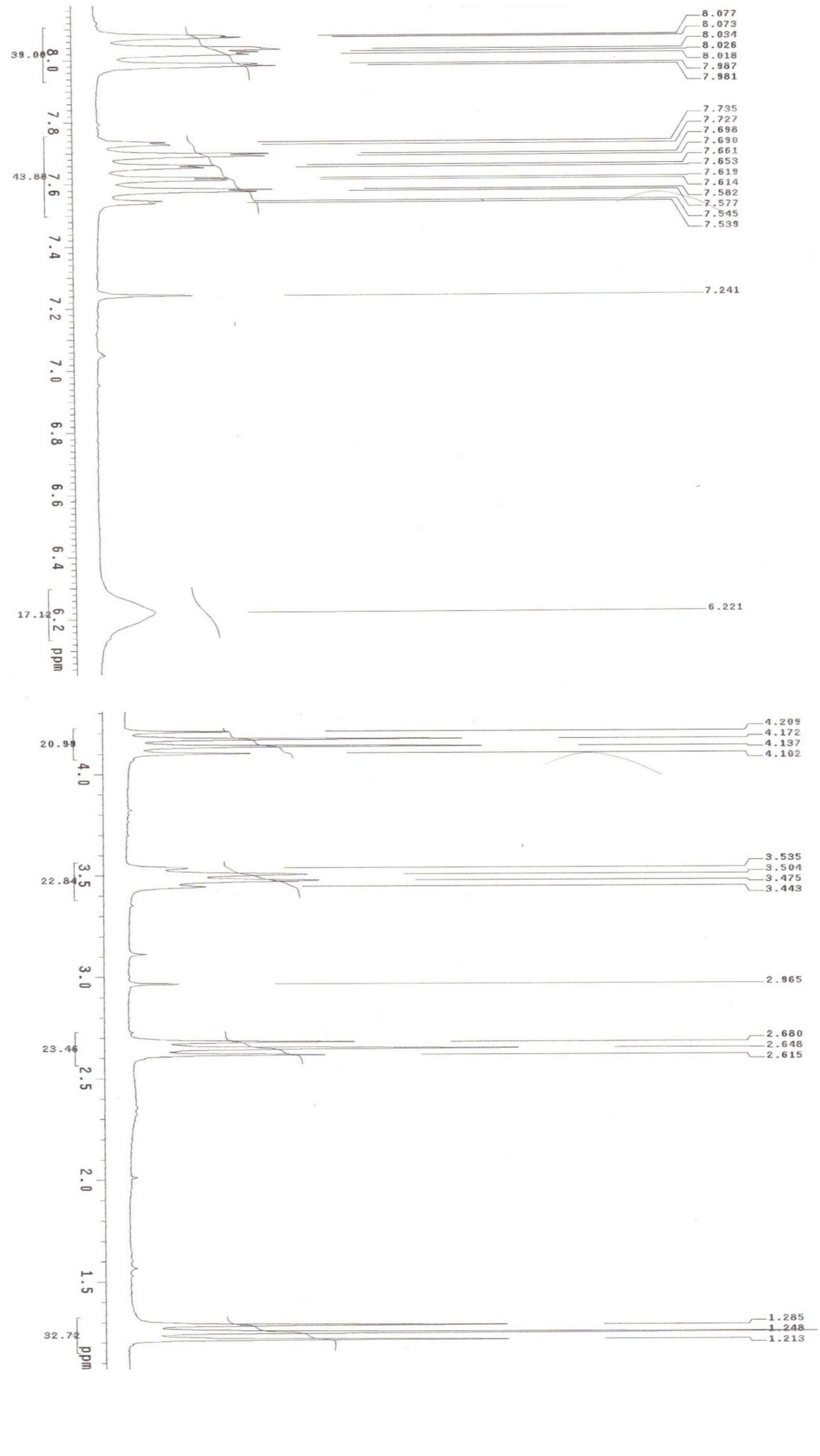

Figure S25. ^{13}C NMR (APT δ, $CDCl_3$, 50 MHz) of **8**.

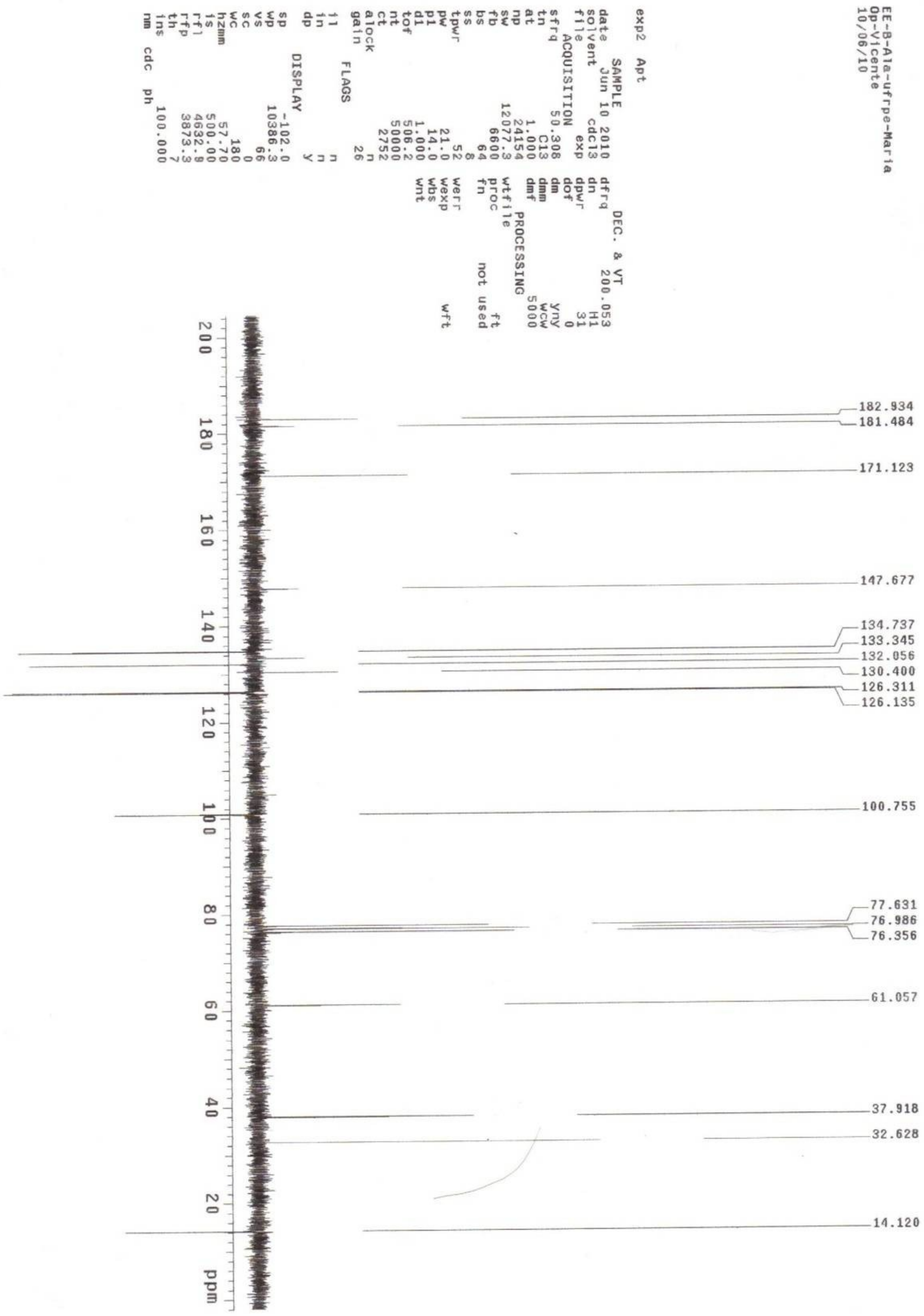

Figure S26. IR (ATR) of **10**.

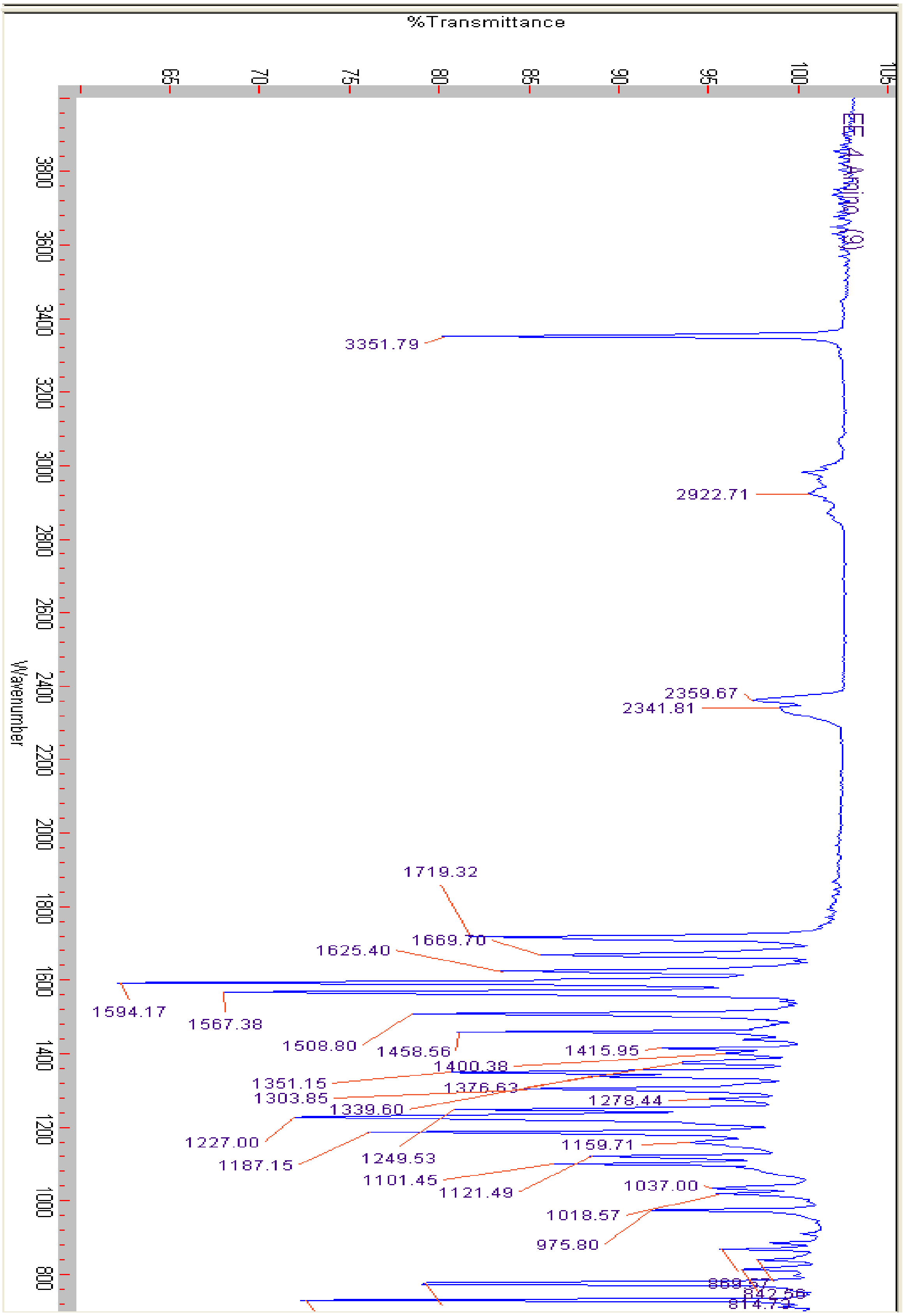

Figure S27. ^{1}H NMR ($CDCl_3$, 200 MHz) and expansions of **10**.

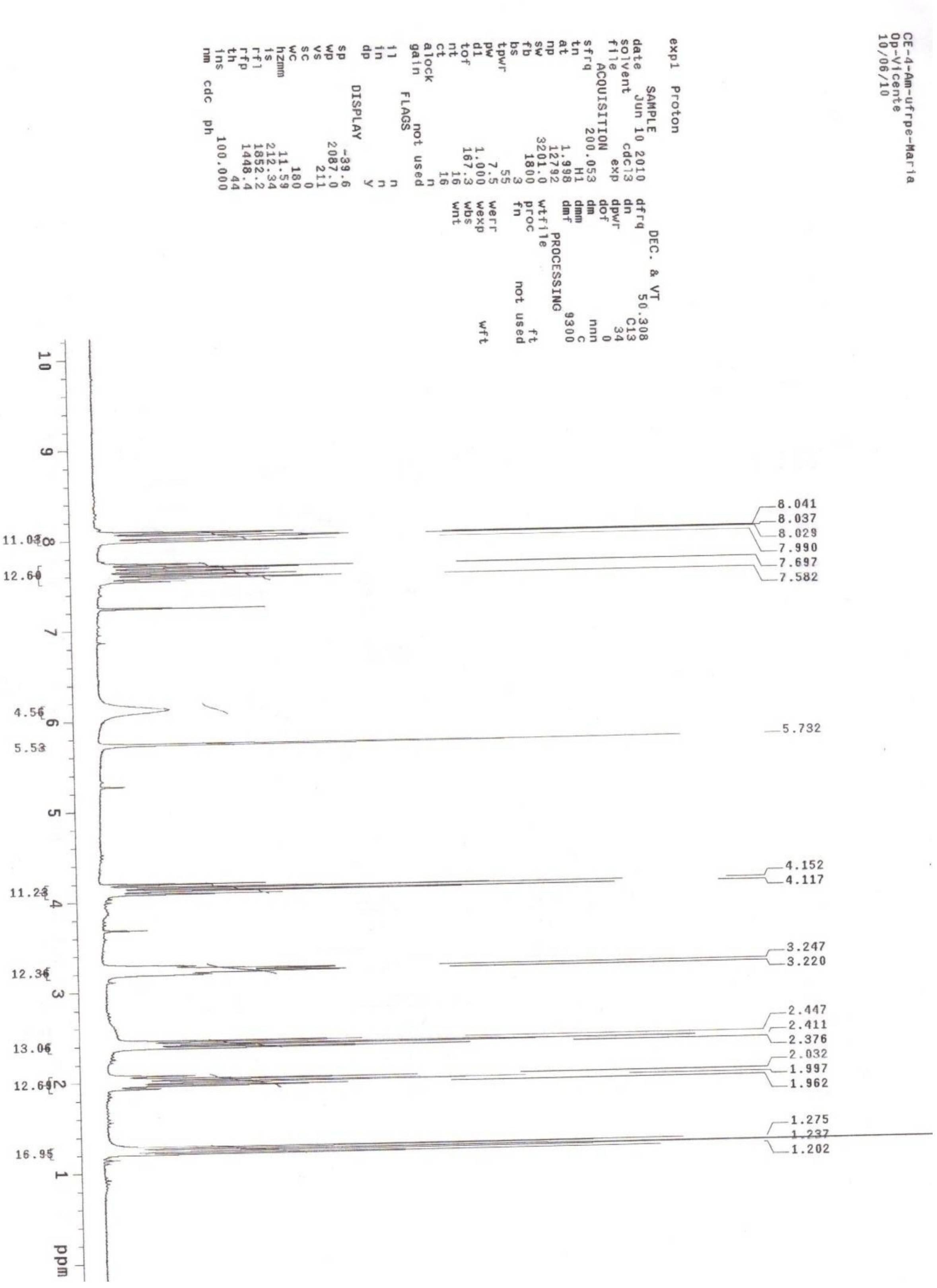

Figure S27. *Cont.*

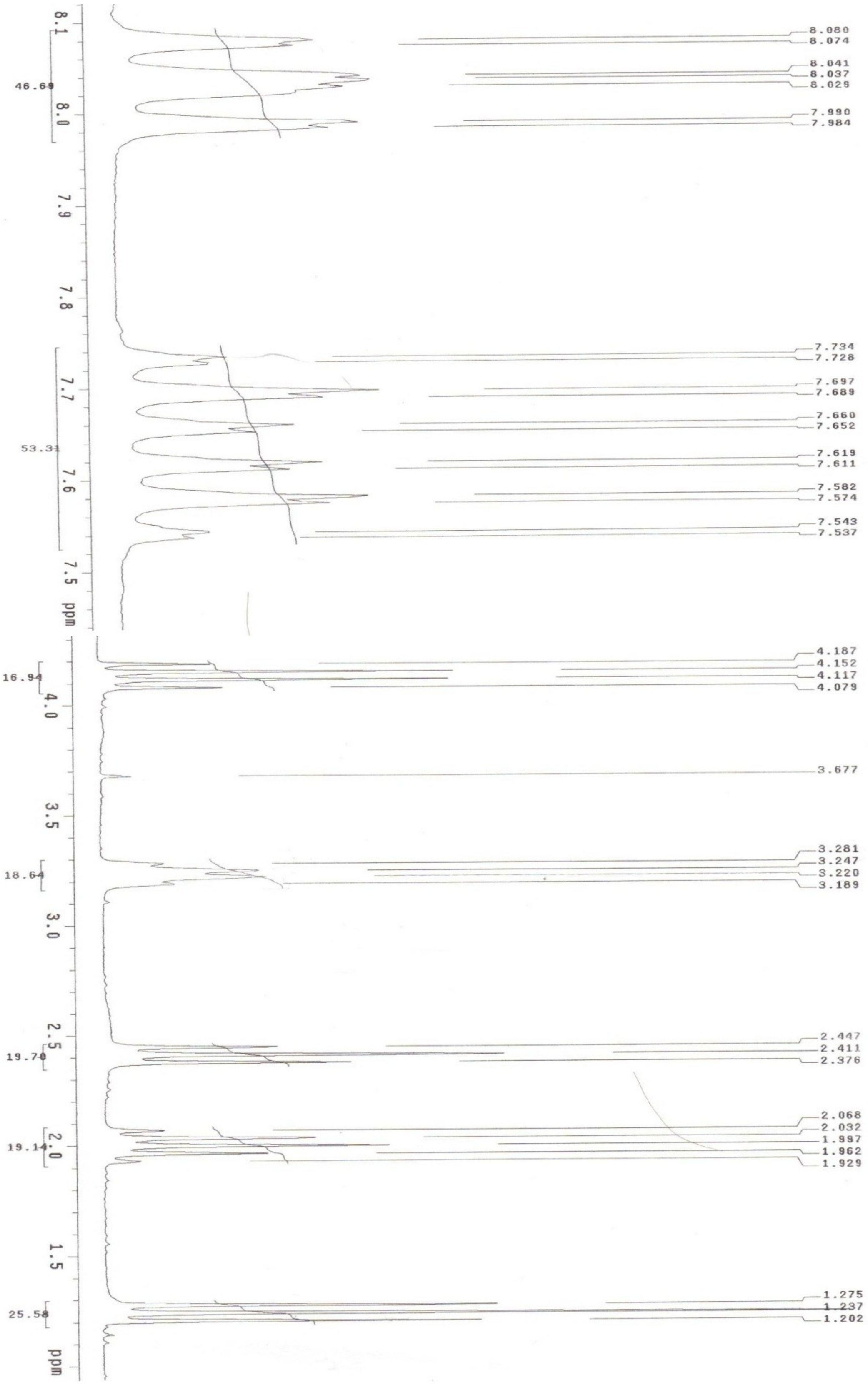

Figure S28. ^{13}C NMR (APT, $CDCl_3$, 50 MHz) of **10**.

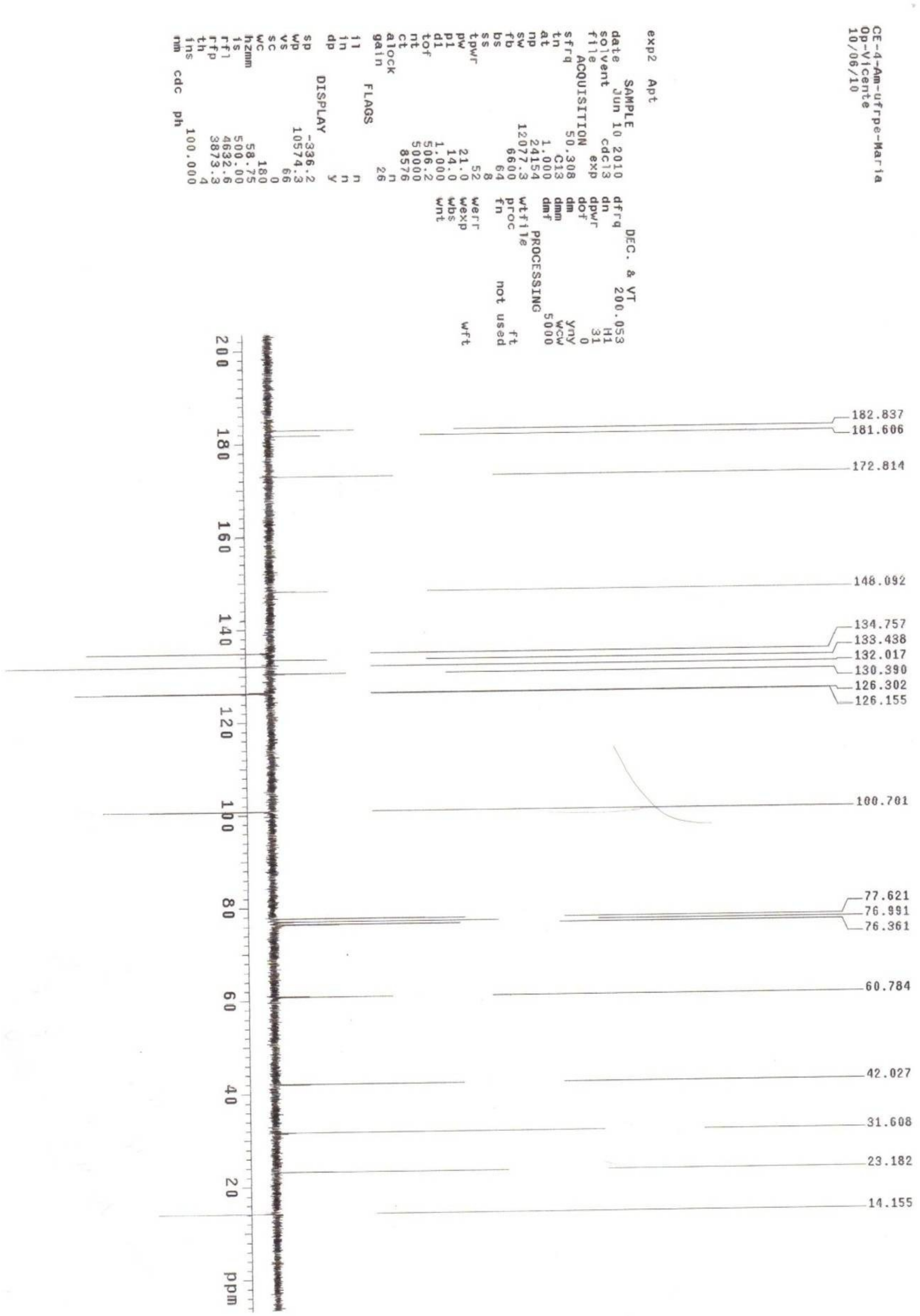

Figure S29. IR (ATR) of **11**.

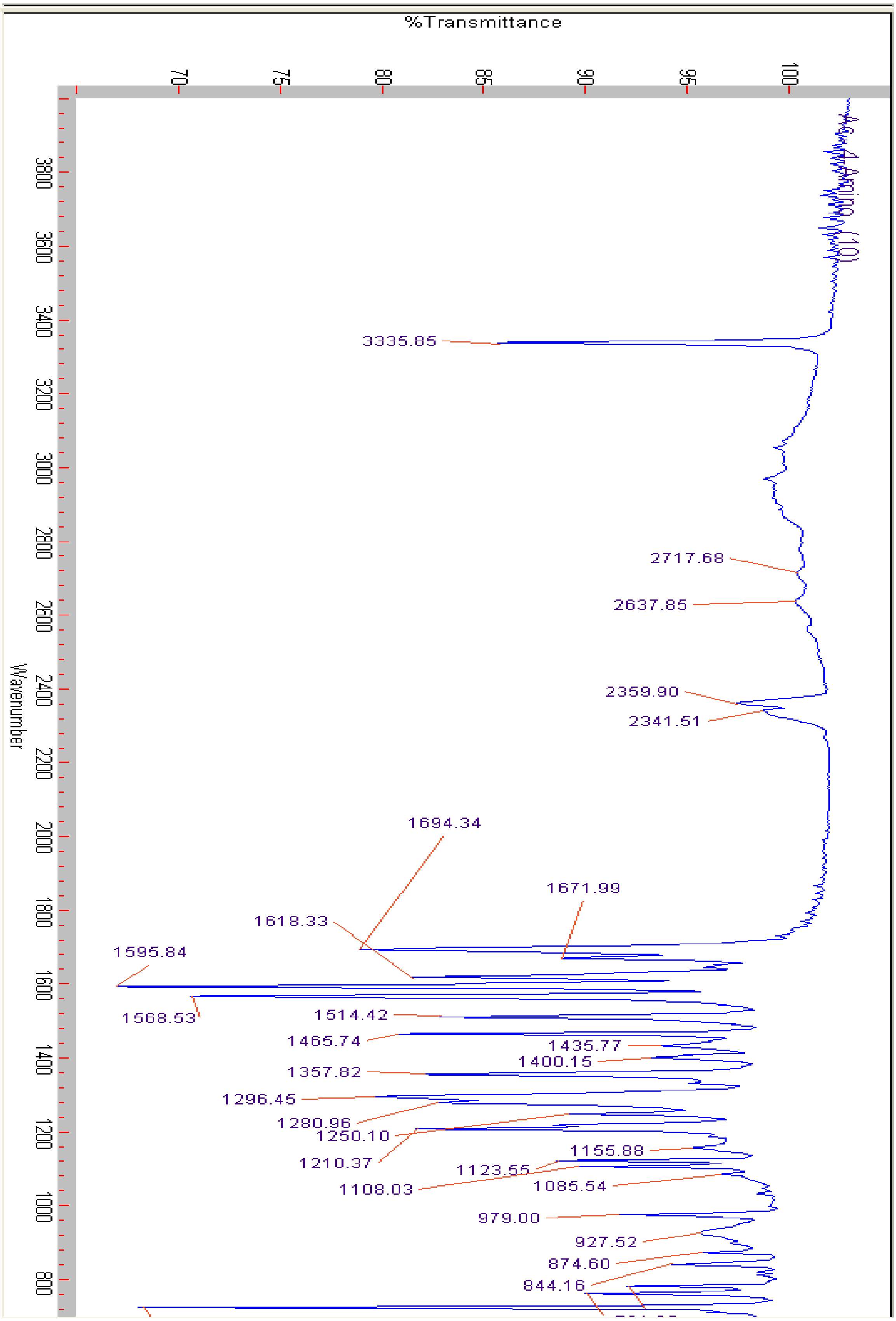

Figure S30. ^{1}H NMR (DMSO-$_{d6}$, 300 MHz) and expansions of **11**.

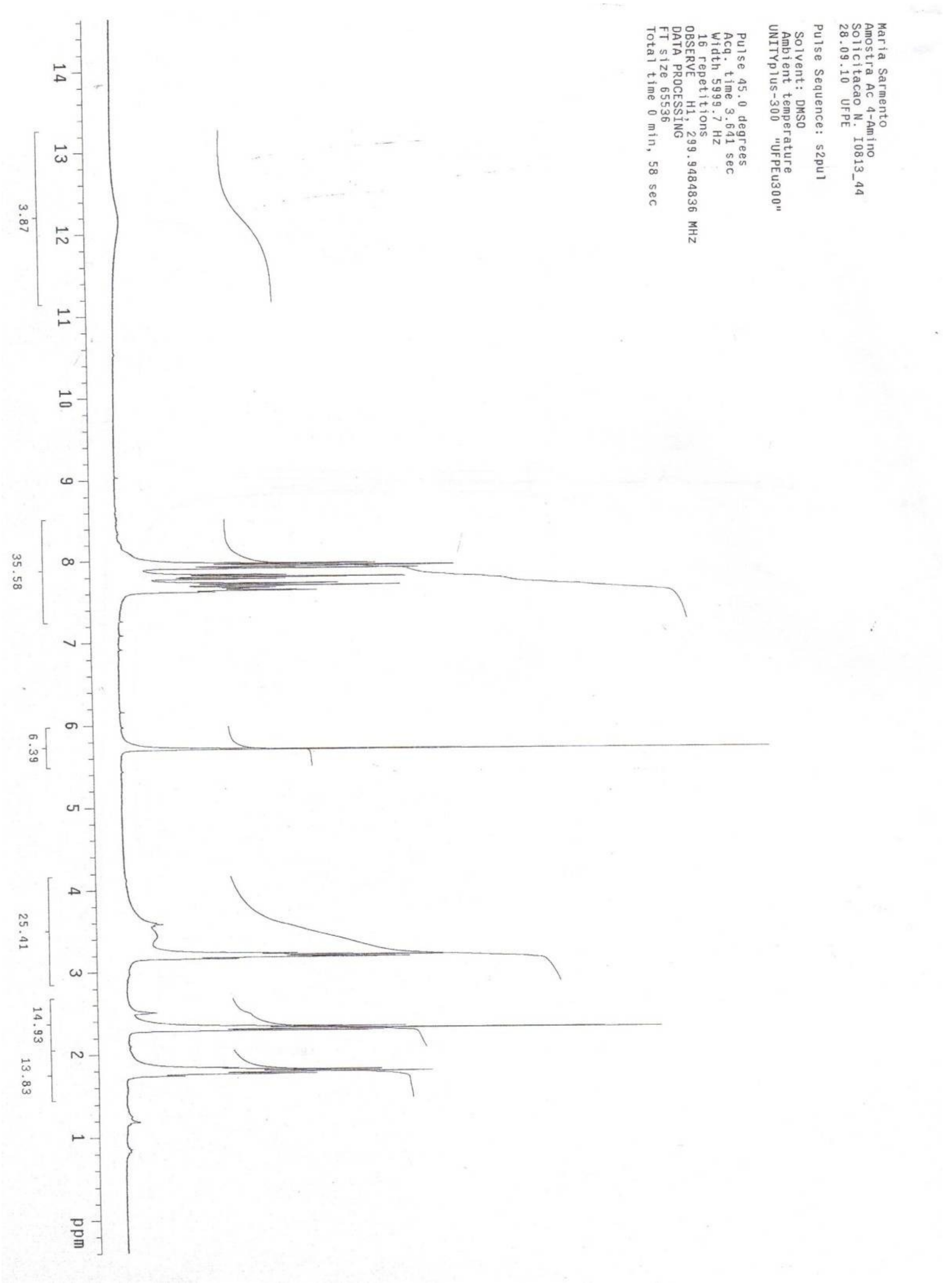

Figure S30. *Cont.*

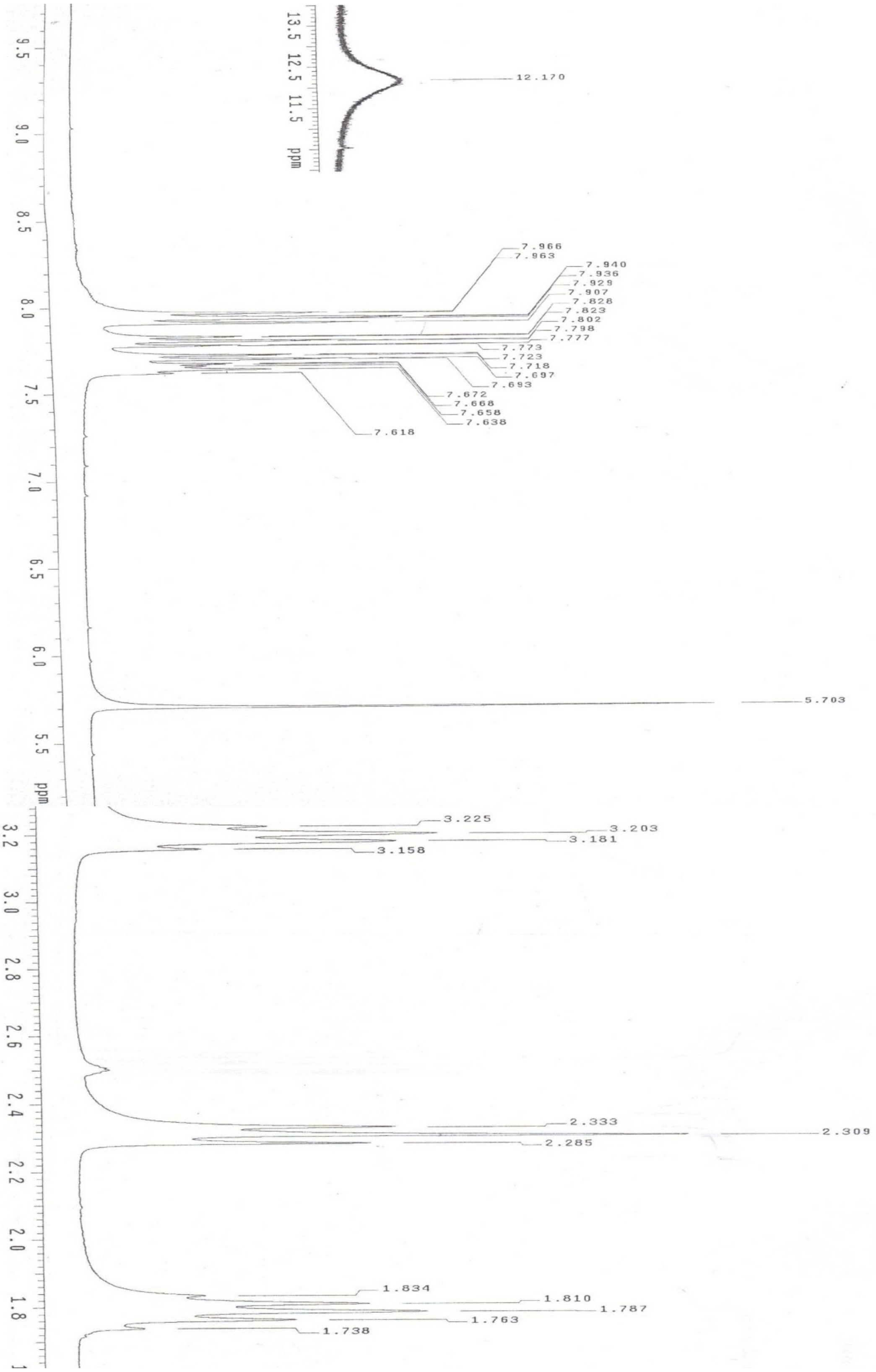

Figure S31. ^{13}C NMR (DMSO-d_6, 75 MHz) and expansions of **11**.

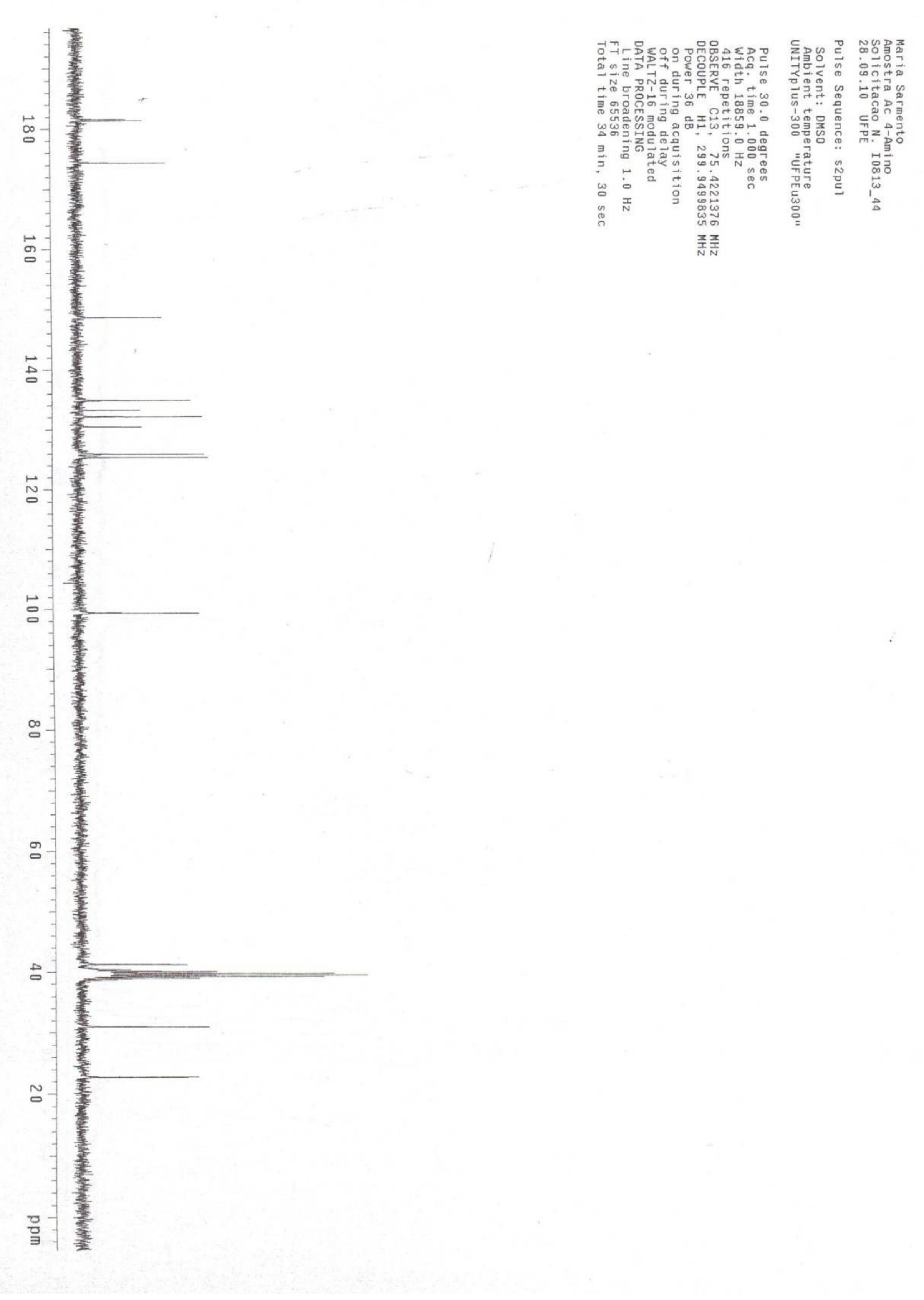

Figure S31. *Cont.*

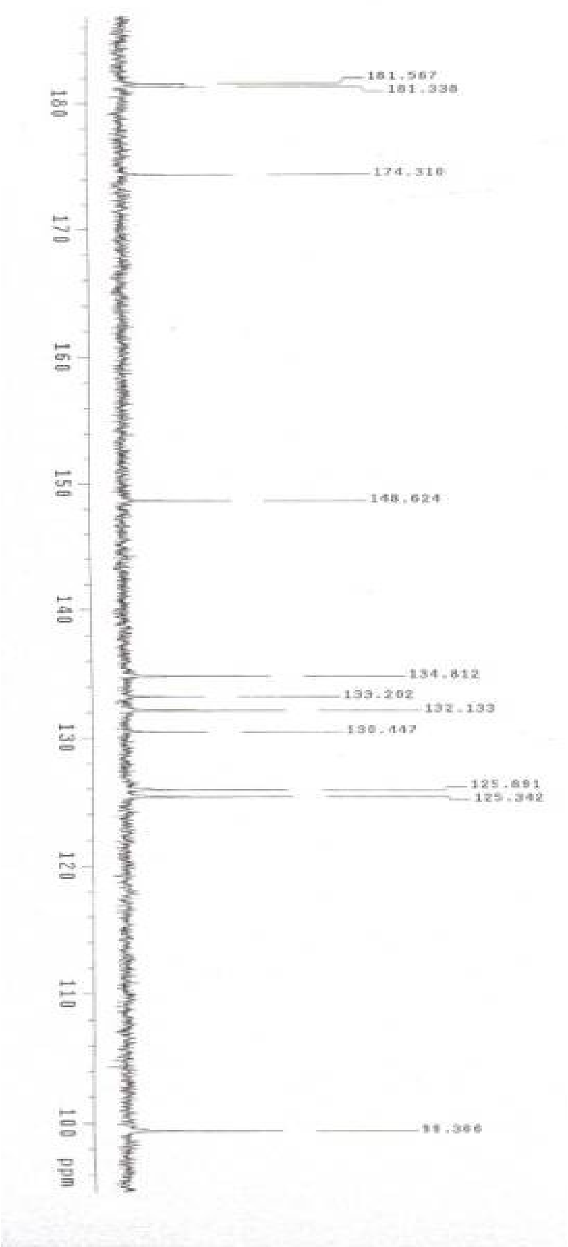

Supplement: Supplementary File 1 [file molecules-19-13188-s001.pdf]
